# Stat3 oxidation-dependent regulation of gene expression impacts on developmental processes and involves cooperation with Hif-1α

**DOI:** 10.1371/journal.pone.0244255

**Published:** 2020-12-17

**Authors:** Michela Grillo, Carolyn Palmer, Nadine Holmes, Fei Sang, Andrew C. Larner, Rahul Bhosale, Peter E. Shaw

**Affiliations:** 1 School of Life Sciences, University of Nottingham, Queen’s Medical Centre, Nottingham, United Kingdom; 2 Deep-Seq Unit, School of Life Sciences, University of Nottingham, Queen’s Medical Centre, Nottingham, United Kingdom; 3 Department of Biochemistry and Molecular Biology, Massey Cancer Center, Virginia Commonwealth University, Richmond, Virginia, United States of America; 4 School of Biosciences, University of Nottingham, Sutton Bonington, United Kingdom; Hokkaido Daigaku, JAPAN

## Abstract

Reactive oxygen species are *bona fide* intracellular second messengers that influence cell metabolism and aging by mechanisms that are incompletely resolved. Mitochondria generate superoxide that is dis-mutated to hydrogen peroxide, which in turn oxidises cysteine-based enzymes such as phosphatases, peroxiredoxins and redox-sensitive transcription factors to modulate their activity. Signal Transducer and Activator of Transcription 3 (Stat3) has been shown to participate in an oxidative relay with peroxiredoxin II but the impact of Stat3 oxidation on target gene expression and its biological consequences remain to be established. Thus, we created murine embryonic fibroblasts (MEFs) that express either WT-Stat3 or a redox-insensitive mutant of Stat3 (Stat3-C3S). The Stat3-C3S cells differed from WT-Stat3 cells in morphology, proliferation and resistance to oxidative stress; in response to cytokine stimulation, they displayed elevated Stat3 tyrosine phosphorylation and *Socs3* expression, implying that Stat3-C3S is insensitive to oxidative inhibition. Comparative analysis of global gene expression in WT-Stat3 and Stat3-C3S cells revealed differential expression (DE) of genes both under basal conditions and during oxidative stress. Using differential gene regulation pattern analysis, we identified 199 genes clustered into 10 distinct patterns that were selectively responsive to Stat3 oxidation. GO term analysis identified down-regulated genes to be enriched for tissue/organ development and morphogenesis and up-regulated genes to be enriched for cell-cell adhesion, immune responses and transport related processes. Although most DE gene promoters contain consensus Stat3 inducible elements (SIEs), our chromatin immunoprecipitation (ChIP) and ChIP-seq analyses did not detect Stat3 binding at these sites in control or oxidant-stimulated cells, suggesting that oxidised Stat3 regulates these genes indirectly. Our further computational analysis revealed enrichment of hypoxia response elements (HREs) within DE gene promoters, implying a role for Hif-1. Experimental validation revealed that efficient stabilisation of Hif-1α in response to oxidative stress or hypoxia required an oxidation-competent Stat3 and that depletion of Hif-1α suppressed the inducible expression of *Kcnb1*, a representative DE gene. Our data suggest that Stat3 and Hif-1α cooperate to regulate genes involved in immune functions and developmental processes in response to oxidative stress.

## Introduction

Mitochondria are essential metabolic organelles in eukaryotic cells and homeostatic mechanisms have evolved to safeguard their function, thereby contributing to the fitness of metazoan organisms [1]. Impaired mitochondrial activity is associated with both increased intracellular ROS and disparate expression of mitochondrial proteins from mitochondrial and nuclear DNA, which are linked to ischaemia-reperfusion injury and premature aging [2, 3]. Hydrogen peroxide generated from mitochondria and dedicated NADPH oxidase (Nox) enzymes via superoxide dismutation can attenuate the activity of cysteine-based enzymes including phosphatases and ROS scavengers, as well as modulating the activity of several transcription factors [4, 5]. It therefore appears that metabolic activity, ROS production and the expression of nuclear genes are mechanistically coupled.

Stat3 is a transcription factor required for stem cell pluripotency and early embryonic development [6, 7]. Its activity in cells is regulated by several cytokines (IL-6, LIF, IL-10) and when tyrosine phosphorylated, Stat3 serves as a transcription factor to stimulate the expression of multiple target genes. However, Stat3 is also reported to activate transcription in a tyrosine phosphorylation-independent manner [8]. In addition to its developmental roles, Stat3 is crucial for cardiac homeostasis, as conditional deletion of Stat3 in the heart perturbed vascularisation and reduced cardiac function in older animals with marked enhancement of fibrosis and markers for programmed cell death (PCD) [9, 10]. Furthermore, ischaemic preconditioning in the heart was shown to require functional Stat3 [11]. The underlying mechanism for these changes is unresolved.

As well as being a nuclear transcription factor, Stat3 operates within mitochondria. It was shown to interact with Grim19 (Ndufa13), a subunit of complex I in the electron transport chain (ETC), and may require this interaction to enter mitochondria [12–14]. In primary cells lacking Stat3, maximal (state 3) oxidation rates and activities of complexes I and II were significantly reduced and re-expression of Stat3 rescued this defect [15]. Stat3 also associates with cyclophilin D, a component of the mitochondrial permeability transition pore (MPTP) complex and could potentially influence mitochondrial respiration by regulating pore opening [16]. The association of Stat3 with cyclophilin D, a component of the MPTP, has been linked to decreased mitochondrial ROS production [17].

Stat3 is also redox-sensitive and we previously identified cysteine residues in its DNA-binding domain (DBD) that are reversibly oxidised in cells treated with peroxide [18]. Substitution of cysteines in the Stat3 DBD created a redox-insensitive mutant Stat3 (Stat3-C3S) and cells that over-expressed Stat3-C3S proliferated more rapidly than cells over-expressing WT-Stat3, although the effect was reversed when ROS levels were elevated [18]. Cysteine oxidation correlated with decreased gene expression from promoters containing a consensus Stat3 binding element (SIE), whereas expression from promoters with a non-consensus SIE was increased [19]. These data implicated Stat3 in responses to oxidative stress and pre-empted the discovery of an oxidative relay between Stat3 and the oxidant scavenger peroxiredoxin II (Prx2) [20]. Moreover, in cardio-specific, heterozygous Grim19 knockout mice, attenuated ischaemia-reperfusion injury was associated with elevated peroxide release from mitochondria, increased expression of Prx2 and formation of Stat3 redox dimers [21].

Here, we describe murine embryonic fibroblasts (MEFs) engineered to express exclusively either WT-Stat3 or Stat3-C3S and their use to determine global gene expression changes associated with Stat3 oxidation. Our findings reveal that Stat3 regulates a set of genes associated with immune function, cell migration, organ development and programmed cell death (PCD) in response to oxidative stress and in cooperation with the Hypoxia-Inducible Factor Hif-1α.

## Materials and methods

### Generation of Stat3^fl/fl^ MEFs

Mice were housed at Virginia Commonwealth University according to guidelines of the Institutional Animal Care and Use Committee who approved this study. Stat3^fl/fl^ embryos were obtained at day 14 of pregnancy. Embryonic tissue was homogenised in PBS and treated with trypsin/DNase I. Cells were collected by centrifugation, re-suspended in DMEM supplemented with 10% FBS and plated at a density of ~2x10^5^/10cm dish.

### Cell culture and immunoblotting

MEFs were cultured in DMEM (Sigma D5671) with 10% FCS, 100 units ml^-1^ penicillin, 100 ug ml^-1^ Streptomycin and 4 mmol L^-1^_L-_glutamine. For immunoblotting from reducing SDS-PAGE gels, standard procedures were used throughout.

### Phalloidin staining

MEFs were seeded onto glass cover slips in 6-well plates at 1.5 x 10^4^ MEFs per well in 2 ml full medium and cultured at 37°C over night. Cells were stained with 165 nM Alexa Fluor 488 phalloidin and imaged at 20x magnification.

### DNA binding assays

Electrophoretic mobility shift assays (EMSAs) for Stat proteins have been described earlier [22].

### Cell proliferation and migration assays

For MTT assays MEFs were seeded in 96-well plates at 250 cells per well in 100 μl full medium and cultured under normal growth conditions for the duration of the assay. Proliferation was measured daily. Alternatively, MEFs were seeded in 6-well plates at 1 x 10^4^ cells per well in 2 ml full medium and grown for up to 120 h under normal conditions and counted daily. For wound healing assays, MEFs were seeded in 10 cm plates and grown under normal growth conditions to confluence. Monolayers were scratched with a 2 μl pipette tip, medium was replaced with fresh full medium and MEFs were photographed at the beginning of the assay (*t* = 0) and after 7 h at 10x magnification using a Leica DMIRE microscope. Images are from a single representative experiment of three biological repeats.

### Cell death assays

MEFs were seeded in 6-well plates at 1 X 10^5^ per well in 2 ml medium and grown for 16 h under normal growth conditions. Cells untreated and treated with various concentrations of peroxide for 6h were trypsinised, collected by centrifugation and re-suspended in 1 ml full medium. Re-suspended cells (100 μl) were added to 100 μl Muse annexin V and dead cell assay reagent and incubated for 20 min at room temperature. Levels of PCD were determined by assessing the percentage of annexin V and propidium iodide positive cells using the Muse Cell Analyzer, collecting 2000 cells per run.

### Gene expression analysis

RNA-sequencing and quantitative RT-PCR are described in the S1 File.

### Chromatin immunoprecipitation (ChIP)

Experiments were performed essentially as described in [18] and analysed by qPCR as described in [23]. ChIP-seq is described in the S1 File.

### Statistical analysis

Tools used to analyse RNA-seq: for qRT-PCR, *post hoc* analyses were conducted using the Sidak (untreated v treated) or Tukey (comparisons between cell types) multiple comparisons test for repeated-measures ANOVA. * P<0.05, ** P< 0.01, *** P< 0.001, **** P< 0.0001.

## Results

### Generation of Stat3 murine cell line model

**Fig 1:**
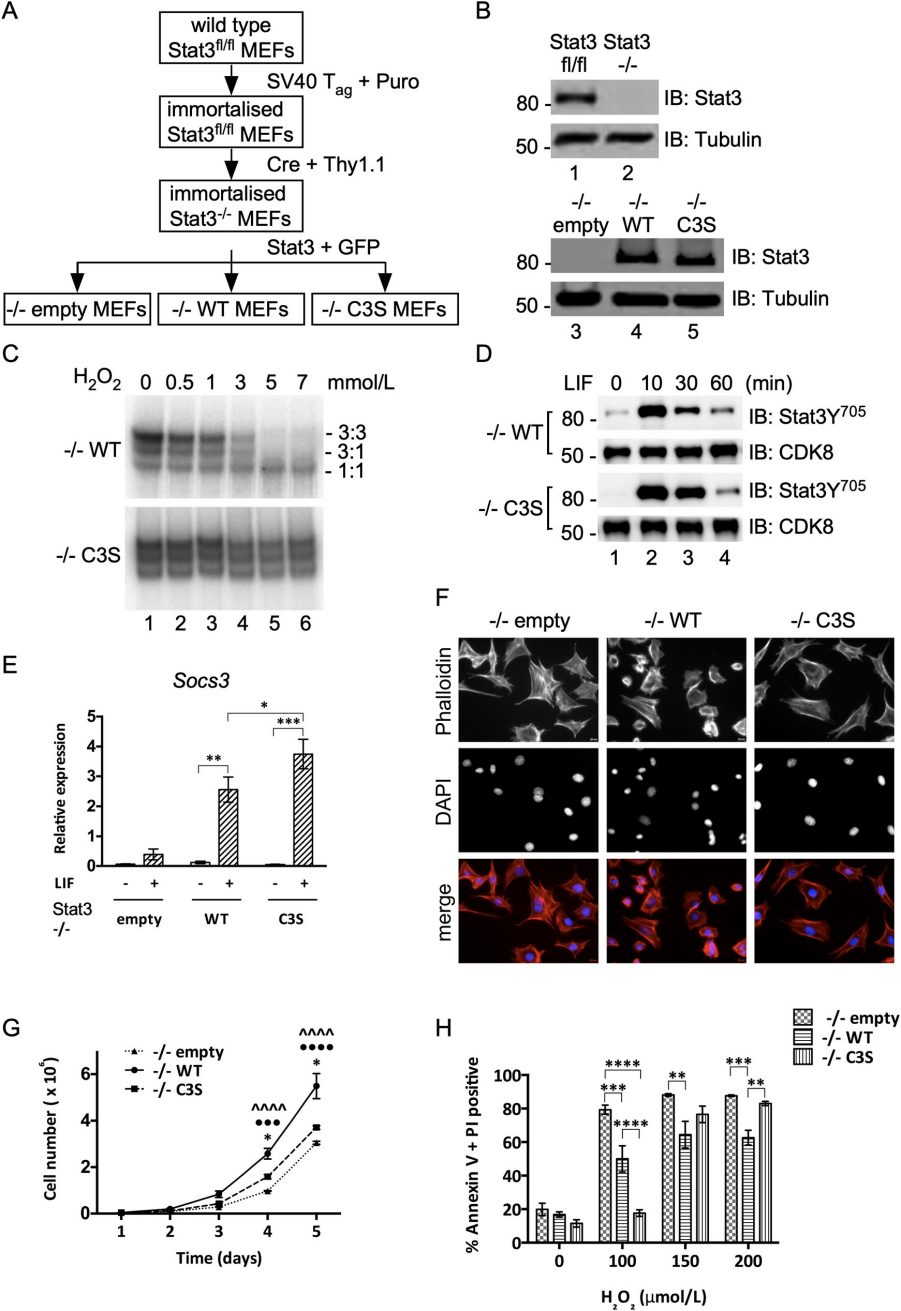
Generation and characterization of Stat3 cell lines. **A)** Flow diagram for the production of Stat3 null (-/-empty), WT-Stat3 (-/-WT) and redox-insensitive Stat3-C3S (-/-C3S) expressing MEFs. **B)** Upper: Immunoblot confirming Stat3 deletion Lower: Immunoblot confirming re-expression of Stat3-WT and Stat3-C3S. **C)** Nuclear extracts from -/-WT and -/-C3S MEFs were incubated with increasing concentrations of peroxide and binding to a radio-labelled m67/SIE was analysed by EMSA. DNA-bound Stat 3:3, 3:1 and 1:1 homo- and heterodimers are indicated. **D)** Induction of Stat3 Y705 phosphorylation in both -/-WT and -/-C3S MEFs upon LIF treatment. Serum starved MEFs were treated with 10ng ml^-1^ LIF and harvested at times indicated. Stat3 Y705 phosphorylation was detected by SDS-PAGE and immunoblotting with an anti-phospho-Y705 antibody. **E)** Induction of *Socs3* expression in -/-WT and -/-C3S MEFs upon LIF treatment. qRT-PCR analysis was performed on *Socs3* and *Hbs1l* mRNA enriched from total RNA from MEFs untreated (-) or treated with 10 ng ml^-1^ LIF for 30 mins (+) using Taqman assays. *Socs3* expression was normalised to *Hbs1l* expression. Data are expressed as mean ± SEM, n = 3. **F)** Phalloidin staining to show the arrangement of actin fibres in -/-empty, -/-WT and -/-C3S MEFs. Cells were stained with 165 nM Alexa Fluor 488 phalloidin and imaged at 20 x magnification. **G)** MEFs were seeded in 6-well plates and grown for up to 120 h under basal conditions. Cells were counted every 24h. **H)** MEFs were seeded and grown for 16 h under normal conditions. Cells were treated with peroxide at the concentrations indicated and levels of PCD were determined using Muse Annexin V and dead cell assay kit after 6 h, collecting 2000 cells per run. Data are expressed as mean ± SEM, n = 3.

Immortalised MEFs from Stat3^fl/fl^ embryos were transduced successively with MSCV vectors for Cre-Thy1.1, and for GFP alone, GFP with WT-Stat3, or GFP with Stat3-C3S. Cells were sorted for Thy1.1 expression to yield Stat3^-/-^ cells and then for GFP expression to derive three polyclonal lines, -/-empty, -/-WT and -/-C3S (Fig 1A). Immunoblotting confirmed the presence or absence of Stat3 expression as expected (Fig 1B). The identities of -/-WT and -/-C3S cells were confirmed by sequencing (not shown) and by measuring the effect of peroxide treatment on Stat3 binding to a consensus SIE (Fig 1C) [18].

LIF stimulated transient phosphorylation of Stat3 on tyrosine 705 (Y705) (Fig 1D) and induced expression of *Socs3*, a well-defined cytokine-inducible Stat3 target gene in -/-WT cells (Fig 1E). In -/-C3S cells, LIF stimulated more robust Y705 phosphorylation and significantly higher levels of *Socs3* expression than in -/-WT cells, confirming that Stat3-C3S is fully competent to mediate cytokine-responsive gene expression. However, -/-empty and -/-C3S cells both showed a flat morphology with long, peripheral actin fibres, unlike -/-WT cells, which appeared smaller and more compact (Fig 1F), suggesting that loss of Stat3 oxidation may impact on cell phenotype.

Cells lacking Stat3 (-/-empty) or expressing Stat3-C3S (-/-C3S) proliferated more slowly than -/-WT cells, a difference substantiated in cell counting and MTT assays (Fig 1G and S1A Fig). Wound-healing assays showed that -/-empty and -/-C3S cells also migrated less quickly than -/-WT MEFs (S1B Fig). These results contrast with earlier findings obtained with cells transiently over-expressing Stat3-C3S [18] that retained an oxidation-competent Stat3 and therefore may not have accurately reflected a phenotype attributable wholly to Stat3-C3S.

To determine resistance to oxidative stress, PCD indices were measured after treatment of the three cell lines with peroxide. Approximately 50% of -/-WT cells became apoptotic 6h after exposure to 100 μM peroxide. In comparison, -/-empty cells were significantly more sensitive, with ~80% of cells apoptotic after 6h, whereas -/-C3S cells appeared refractory to 100 μM peroxide (Fig 1H and S1C Fig). At super-physiological levels of oxidant stress (150 μM and 200 μM peroxide) all three cell types exhibited high levels of apoptosis, although WT-Stat3 provided a significant level of protection (Fig 1H). Thus -/-empty cells are hypersensitive to oxidative stress, whereas -/-C3S cells appear to be de-sensitised.

### Impact of Stat3 oxidation on gene expression

**Fig 2:**
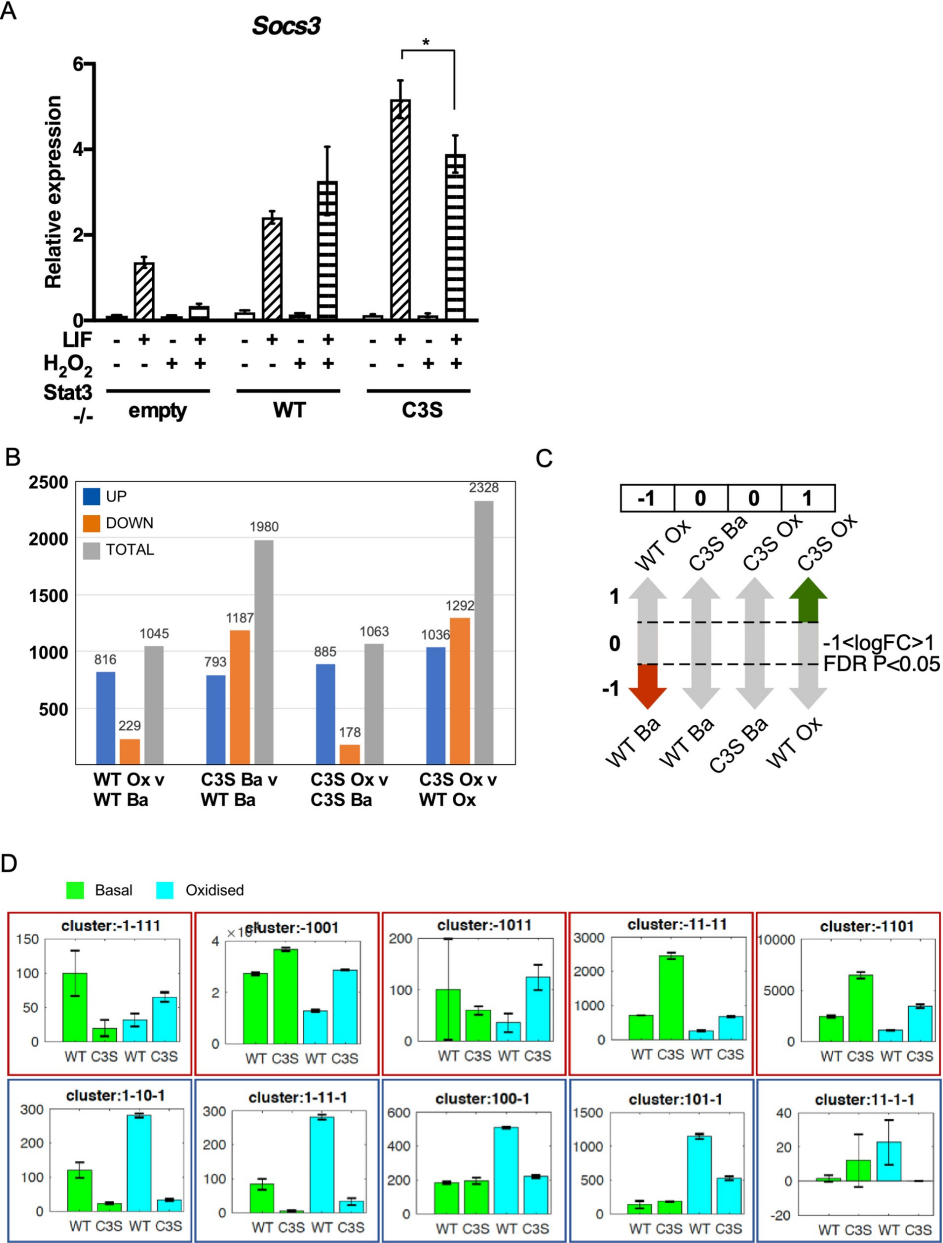
Differential -/-WT and -/-C3S expression profiling reveals genes acutely responsive to Stat3 oxidation. **A)** RNA isolated from MEFs untreated (-), treated with 10 ng ml^-1^ LIF for 30 mins and/or 100 μM H_2_O_2_ for 1 h (+) was enriched for mRNA and analysed for *Socs3* and *Hbs1l* expression by qRT-PCR. *Socs3* expression was normalised to *Hbs1l*. Data are expressed as mean ± SEM, n = 3. **B)** Bar graph showing number of differentially regulated genes per comparison. **C)** Rationale for discretisation of DE genes from four-way comparison into clusters characterised by distinct multivariate expression patterns. Significant DE in each comparison (e.g. -/-WT basal v oxidation) scores 1 (increase), -1 (decrease) or 0 (no significant change). Diagram indicates DE pattern characteristic for cluster -1,0,0,1. **D**) Bar graphs showing a random example for each of 10 unique patterns identified from 3617 differentially expressed genes. Y-axes show normalised counts. Blue and red outlines indicate Stat3 oxidation-responsive gene expression significantly up- or down-regulated, respectively, in control v oxidation -/-WT cells and dis-regulation in -/-C3S cells.

Oxidation of cysteines in Stat3 inhibited binding to consensus SIEs and decreased Stat3-mediated gene expression [18, 20]. Thus, we first investigated the effect of oxidative stress on cytokine-dependent *Socs3* expression in all three cell lines. Unexpectedly, peroxide treatment did not decrease LIF-induced *Socs3* expression in -/-WT cells, although it decreased LIF-induced *Socs3* expression in -/-empty and -/-C3S cells (Fig 2A), implying that ROS-sensitive components other than Stat3 participate in regulating *Socs3* expression. This observation also suggested that Stat3 oxidation could have a positive impact on the expression of some genes and contribute to distinct gene expression profiles in -/-WT and -/-C3S cells.

To understand how Stat3 oxidation affects gene expression globally during oxidative stress, we performed RNA-sequencing of -/-WT and -/-C3S cells under control and peroxide treatments. Two biological replicates were collected for each cell line per treatment. In total, >260 million high quality reads were generated using the Illumina Hiseq 2500 sequencing platform and mapped to the mm10 reference genome using software Tophat2 v2.0.10. An average 60% of reads were uniquely mapped to exons of annotated murine genes (S1 Table) and only the uniquely mapped reads were used for further analysis. Raw reads were normalised and differential expression was performed using MATLAB’s RNAseq data analysis pipeline (https://uk.mathworks.com/help/bioinfo/ug/identifying-differentially-expressed-genes-from-rna-seq-data.html). To reduce the influence of transcription noise, we defined a gene as expressed if its maximum read counts (averaged over two replicates) across all profiles was ≥10. In total, 3617 genes were identified to be differentially expressed (*P* < 0.05, Benjamini-Hochberg FDR correction and -1<logFC>1) between comparisons of -/-WT vs -/-C3S cells and basal vs oxidation treatments (S2 Table). Similar numbers of genes were differentially expressed between oxidation v basal in both -/-WT and -/-C3S cells. However, more genes were differentially expressed between -/-WT vs -/-C3S cells under basal conditions and even more after oxidative stress (Fig 2B).

To identify patterns among DE genes, we discretised gene regulation information of each gene from four pairwise comparisons into -1 (down-regulated), 1 (up-regulated) or 0 (un-regulated). For instance, in gene cluster -1,0,0,1, the first position attributes to comparison of oxidation v basal in -/-WT cells, the second position to comparison of -/-C3S v -/-WT cells under basal conditions, the third position to oxidation v basal in -/-C3S cells and the fourth position attributes to comparison of -/-C3S v -/-WT cells post oxidation (Fig 2C). This approach identified 45 distinct gene clusters (S2A Fig), from which we selected 10 clusters that showed Stat3 oxidation-responsive gene expression i.e. significant up- or down-regulation in oxidation v basal in -/-WT cells and dis-regulation in -/-C3S cells in other comparisons, as exemplified in Fig 2D. These 10 clusters contain a total of 199 genes with 86 genes down-regulated and 113 up-regulated in response to peroxide treatment of -/-WT cells (S3 Table).

The finding that a majority of DE genes was up-regulated in -/-WT cells after acute peroxide stimulation was unanticipated so we performed qRT-PCR experiments on several representative genes, including *Aplnr* (angiotensin II-like Apelin receptor), *Asprv1* (aspartic protease TAPS), *Il17f* (Interleukin 17) and *Kcnb1* (rectifying potassium channel Kv2.1) [24–27]. In -/-WT cells stimulated with peroxide, expression of these four genes was induced 10-45-fold, whereas induction in -/-C3S and -/-empty cells was substantially lower in all cases (S2B–S2E Fig), confirming that oxidation of Stat3 contributes to their acute up-regulation during oxidative stress. We also determined the expression of several genes assigned as non-DE, such as succinate dehydrogenase subunit B (*Sdhb*) and NADH dehydrogenase Fe-S protein 6 (*Ndufs6*), or excluded from our clusters of interest, e.g. tumour suppressor p53 (*Trp53*), confirming their unresponsiveness to oxidative stress (S2F–S2H Fig). Even so, basal expression of these genes appeared lower in cells lacking an oxidation competent Stat3.

### Biological roles of genes responsive to Stat3 oxidation

**Fig 3:**
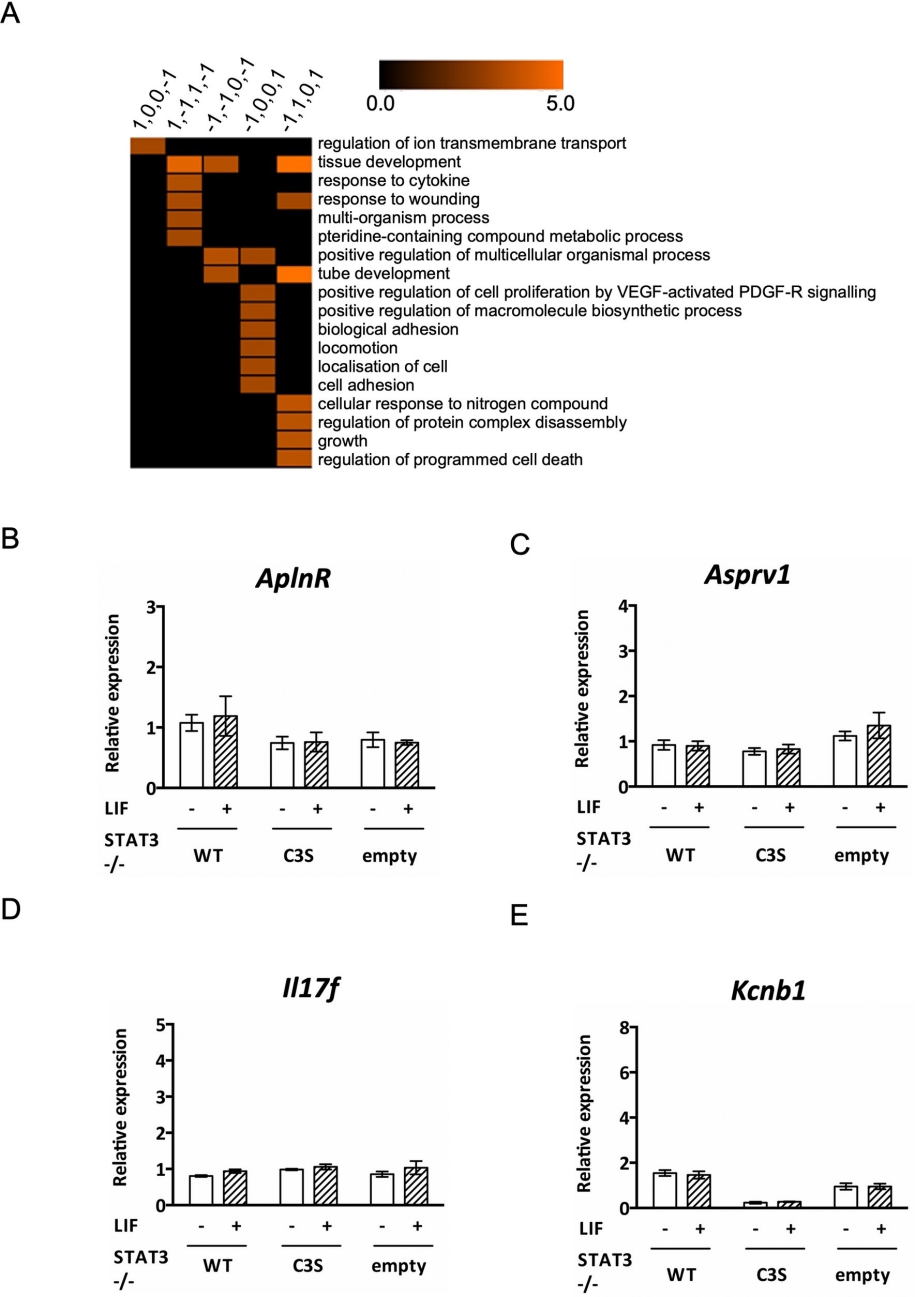
GO enrichment of clusters that showed Stat3 oxidation-responsive gene expression. **A)** Pruned version of total GO enrichment for 5 (out of 10) expression clusters displayed in Fig 2D (the remaining five clusters do not show enrichment for any GO term). GO enrichments obtained with BiNGO [28] were pre-filtered using REVIGO (http://revigo.irb.hr) [29] to remove semantically redundant GO terms. For each cluster, terms were first pruned on REVIGO frequency (>0.1% and <1%), and only the top 5 remaining terms with the lowest REVIGO dispensability were visualized. Full GO enrichment results can be found in S6 Table. Only cells with corrected *P* ≤ 0.05 of GO enrichment are coloured and colour bar indicates–log_2_(p-value). **B-E)** qRT-PCR analysis was performed on genes indicated using mRNA enriched from total RNA from MEFs untreated (-) or stimulated (+) with 10 ng ml^-1^ LIF for 30 min. Expression of all genes was normalised to expression of *HBS1L*. *Post hoc* analyses were conducted using the Sidak (untreated v treated) or Tukey (comparisons between cell types) multiple comparisons test for repeated-measures ANOVA. ** P< 0.01, **** P< 0.0001. Data are expressed as mean ± SEM, n = 3.

GO enrichment analyses of the 199 up- and down-regulated genes in -/-WT cells (and dis-regulated in -/-C3S cells) showed that in general, up-regulated genes are enriched in regulation of cell-cell adhesion, immune responses and transport related processes, while down-regulated genes are primarily enriched in processes related to tissue and organ development and morphogenesis (S4 and S5 Tables). Moreover, GO enrichment analysis of individual gene clusters showed further granular enrichment (S6 Table). For example, cluster 1,0,0,-1 (genes up-regulated in -/-WT cells upon peroxide treatment v control but down-regulated in -/-C3S v -/-WT cells under peroxide treatment) is strongly associated with genes linked to membrane transport related processes. In comparison, cluster -1,0,0,1 (genes down-regulated in -/-WT cells upon peroxide treatment v control but up-regulated in -/-C3S v -/-WT cells under peroxide treatment) is enriched for cell migration and adhesion (Fig 3A).

We note that 22/199 genes are annotated to cell migration (14 to cell migration and 11 to positive regulation of cell migration; 3 overlapping between two GO categories). The majority of these genes are down-regulated in response to oxidative stress in -/-WT cells, whereas in -/-C3S cells their expression remains relatively unchanged or with expression comparable to that in unstimulated -/-WT cells, which suggests that oxidation of Stat3 correlates with their repression (S3A and S3B Fig). Conversely, expression of those genes that are up-regulated in -/-WT cells in response to oxidation, such as *Icam1*, *Cd274*, *Foxj1*, is abrogated in -/-C3S cells.

A similar number of DE genes (25/199) is annotated to regulation of PCD and apoptotic process (S3C Fig). Regardless of being up- or down-regulated in -/-WT cells, their expression in -/-C3S cells is insensitive to oxidative stress, which is consistent with the lower rate of apoptosis seen in -/-C3S cells compared to -/-WT cells (Fig 1H). The majority of the down-regulated genes also appear to be involved in developmental processes (Fig 3A).

Oxidation-dependent gene down-regulation could be due to loss of Stat3 DNA binding, as proposed earlier [18, 20], so we scanned promoter regions (3kb) of DE genes for Stat3 binding sites corresponding to the SIE consensus (TTCCNGGAA) and found that 81/86 down-regulated genes have Stat3 binding sites (up to 2 mismatches) in their promoters (S7 Table). Of these 81 genes, 55 had lower basal expression in -/-WT cells compared to -/-C3S cells, which express a redox-insensitive Stat3. Moreover, 46/55 genes were not significantly down-regulated in -/-C3S cells under oxidative stress, implying that a Stat3-dependent oxidant-responsive mechanism limits their expression. However, motif searching within the promoter regions of up-regulated DE genes revealed that 95/113 genes, including *Aplnr*, *Asprv1*, *Il17f* and *Kcnb1*, also have Stat3 consensus motifs (up to 2 mismatches) in their promoters (S7 Table). Nonetheless, these genes were not stimulated by LIF in any of the cells (Fig 3B–3E), excluding them as novel cytokine-inducible Stat3 genes. The presence of consensus SIEs in many DE gene promoters suggested that Stat3 could regulate their expression directly in response to oxidative stress.

### Stat3 binding is absent from oxidative stress response gene loci

To explore the relevance of consensus SIEs in the promoters of DE genes, we used chromatin immunoprecipitation (ChIP) assays to determine Stat3 DNA occupation in -/-WT cells under different conditions. First, we confirmed Stat3 binding to the *Socs3* and *Fos* promoters in cells treated with LIF to validate the assay conditions (S4A and S4B Fig). Peroxide treatment did not stimulate Stat3 binding to either promoter but appeared to decrease binding in response to LIF, albeit not significantly, in line with earlier reports [18, 20]. These results confirmed that both agonists influence Stat3 DNA binding.

Next, we sought to detect Stat3 binding upstream of *Kcnb1*, a validated DE gene (cluster 1,0,0,-1) induced in response to peroxide stimulation only in -/-WT cells (S2E Fig) but unresponsive to LIF (Fig 3E). Murine *Kcnb1* is expressed as multiple transcripts from two promoters, designated P1 and P2 [30]. The P2 promoter has a consensus SIE at -217; it also harbours multiple copies of the motif AGGxxxAGG, with which Stat3 mutants linked to Hyper IgE Syndrome (HIES) have been proposed to make non-canonical contacts [31]. We used 6 primer sets to scan this+

**Fig 4:**
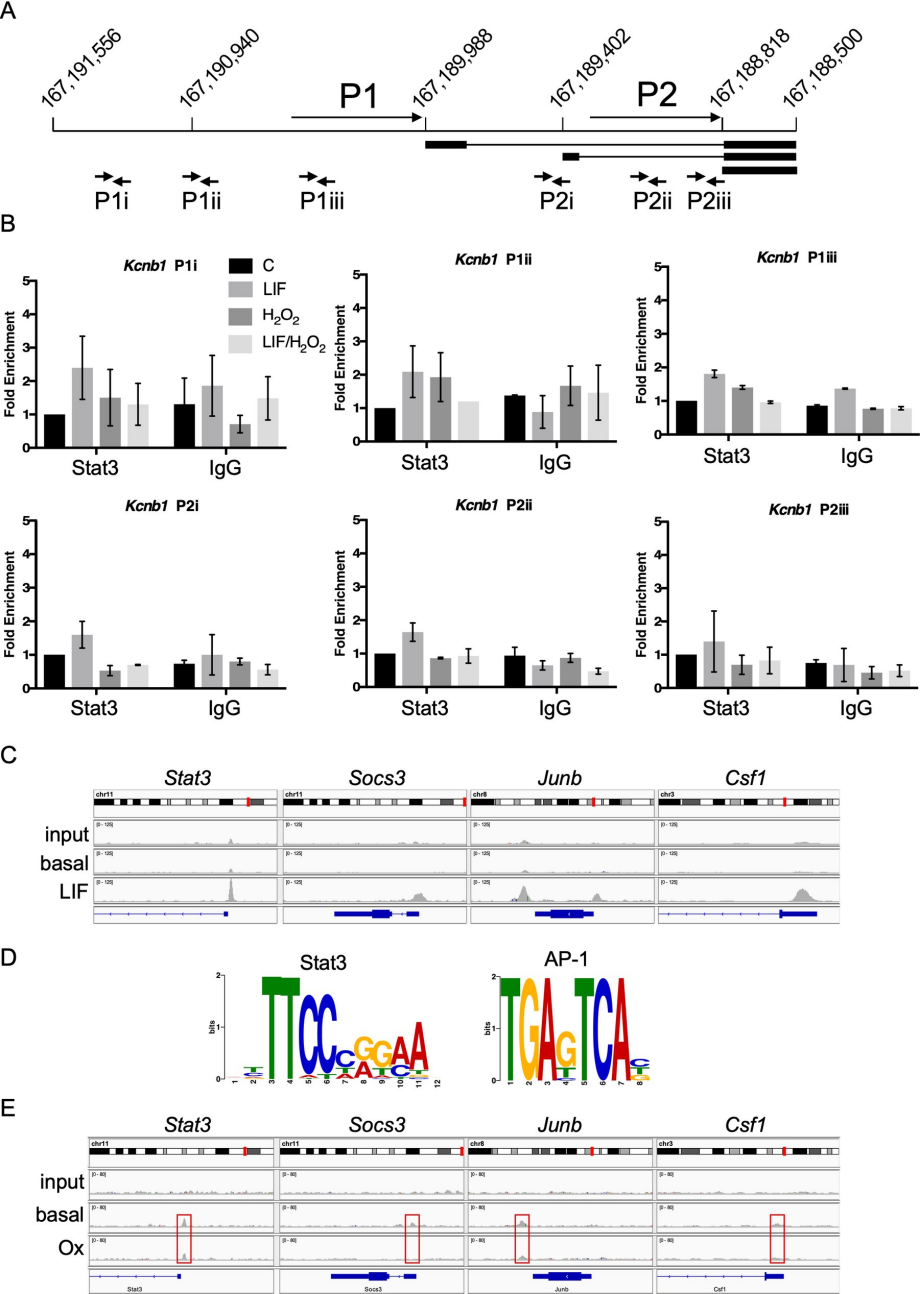
Oxidant-response genes are not associated with Stat3 chromatin binding. **A)** Diagram of murine *Kcnb1* gene promoter region on chromosome 2 with coordinates above horizontal line. Transcripts from P1 and P2 promoters are indicated with exons as bars and introns as lines. Short arrows below transcripts show location of primers pairs used in ChIP assays for Stat3 binding. **B)** ChIP analysis of Stat3 binding across *Kcnb1* promoter region with primer pairs shown in (A) in unstimulated -/-WT MEFs or treated with LIF (10 ng ml^-1^), peroxide (100 μM) or both. Error bars show SEM, n = 3 throughout. **C)** ChIP-seq analysis of LIF-inducible Stat3 binding to promoters of canonical target genes *Stat3*, *Socs3*, *Junb* and *Csf1* in -/-WT MEFs. Images from IGV show chromosomal locations in red (upper), read peaks in grey (middle) and gene organisation in blue (lower track). **D)** Transcription factor binding motifs enriched within -/+ 80 bp of Stat3 read peaks from LIF-stimulated -/-WT MEFs determined by MEME motif discovery software. **E)** ChIP-seq analysis of Stat3 binding to promoters of canonical target genes *Stat3*, *Socs3*, *Junb* and *Csf1* in -/-WT MEFs under basal conditions or after peroxide stimulation (100 μM). Image layout as in (C).

~2.8kb region (Fig 4A) but found no evidence of Stat3 binding in -/-WT MEFs following LIF or peroxide treatment (Fig 4B). Consistent with this result, P1 and P2 promoter fragments of ~1kb were unresponsive to peroxide stimulation in luciferase reporter assays performed in -/-WT and -/-C3S cells (S4C Fig). Thus, regulatory elements crucial for an oxidative response appear to lie outside this *Kcnb1* promoter region.

We then performed ChIP-seq analyses to determine global patterns of Stat3 chromatin binding in -/-WT and -/-C3S cells under different conditions. DNA was immunoprecipitated with anti-Stat3 or control IgG antibodies, as before (S4A and S4B Fig), from cells stimulated with LIF or peroxide or from untreated controls. For each condition, libraries were prepared and sequenced from two independent ChIP replicates. Approx. 8x10^6^ unique reads were obtained per library (2.2x10^7^ for LIF-stimulated samples) and mapped to the mm10 genome assembly.

In unstimulated -/-WT cells, we detected 230 Stat3 binding peaks with a read depth more than 2-fold above IgG controls. The selected read peaks were distributed within intergenic regions and introns. By comparison, in LIF-stimulated -/-WT cells we observed 1088 Stat3 binding peaks that were >2-fold above IgG controls. A significant proportion of these read peaks (20%) mapped to gene promoters (-5kb to +0.5kb from annotated transcriptional start sites) (S4D Fig), including those of known Stat3 targets such as *Stat3*, *Socs3*, *Junb* and *Csf1* (Fig 4C). Using MEME motif discovery software to scan sequences -/+80bp from read peaks we found that the consensus SIE was highly enriched (1056 sites, 4.1e-557); the only other binding motif to show enrichment was an AP-1 consensus (241 sites, 8.4e-49) (Fig 4D). These data confirmed the reliability of the Stat3 antibody and our ChIP-seq procedures.

Read peaks from LIF-stimulated -/-WT cells were associated with 927 genes potentially corresponding to LIF-inducible Stat3 targets. To test their validity as Stat3 target genes, we compared them against alternative Stat3 target gene sets. Numerous studies have sought to identify Stat3 target genes and their relation to biological events influenced by Stat3 [32–40]. These studies used Stat3 activation by cytokines (LIF, oncostatin M) or expression of Stat3C, a hyperactive Stat3 protein with increased DNA binding affinity and resistance to de-phosphorylation [22, 41]. Data from all of the above studies was amalgamated into a single, canonical Stat3 target gene set (S7 Table). We also used data from a ChIP-seq study to determine genome-wide *in vivo* Stat3 binding loci and define genes regulated by Stat3 in murine embryonic stem cells (mESCs) [42]. Hypergeometric tests revealed that the 927 genes we identified overlap significantly with the Stat3 canonical (2.77e-4) and ESC (3.28e-15) gene sets (S4E Fig), but there was no significant overlap with the set of oxidant-responsive DE genes.

A different picture emerged from ChIP-seq analysis of cells treated with peroxide. Overall, read peaks were less well defined than those from LIF-stimulated -/-WT MEFs and the majority (ca. 80%) mapped to intergenic regions (S4D Fig). Peak calling and thresholding implied that ~250 peaks were present only in peroxide-stimulated -/-WT cells, yet these peaks were clearly present in peroxide-stimulated -/-C3S MEFs and unstimulated controls, though not in -/-WT cells stimulated with LIF (S4F Fig). None mapped to DE gene promoters. Thus, it appears that oxidant stimulation does not induce Stat3 chromatin association whereas LIF treatment induces significant *de novo* binding to consensus SIEs. The impact of peroxide on Stat3 in these experiments was nonetheless apparent, insofar as basal Stat3 binding detected in -/-WT controls was decreased after peroxide treatment (Fig 4E). Regulation of oxidative stress responsive genes therefore appears unlikely to involve Stat3 acting directly at the level of chromatin.

### Stat3 cooperates with Hif-1α to regulate *Kcnb1* in response to oxidative stress

In the absence of Stat3 binding at DE gene promoters, we considered whether transcriptional responses to Stat3 oxidation might be mediated indirectly through other factors. To this end, we scanned the 3kb promoter regions of the 199 DE genes for binding motifs of other transcription factors implicated in oxidative stress responses, including cJun, Nrf2 (Keap1) and Hif-1α [43–45]. Our search criteria returned 98/199 canonical AP-1 sites (cJun; S8 Table), 152/199 HREs (Hif-1α; S9 Table) and 58/199 AREs (Nrf2; with up to 2 mismatches; S10 Table). However, there was no apparent relationship between the presence of any of these binding motifs, or combination thereof, and pattern of DE gene regulation. Therefore, because 75% of DE gene promoters appeared to contain HREs and prompted by previous reports of a functional relationship between Stat3 and Hif-1α [46, 47] a role for Hif-1α seemed plausible. To explore this possibility, we focused on *Kcnb1* [48, 49], a DE gene acutely stimulated by peroxide only in -/-WT cells and unresponsive to LIF.

**Fig 5:**
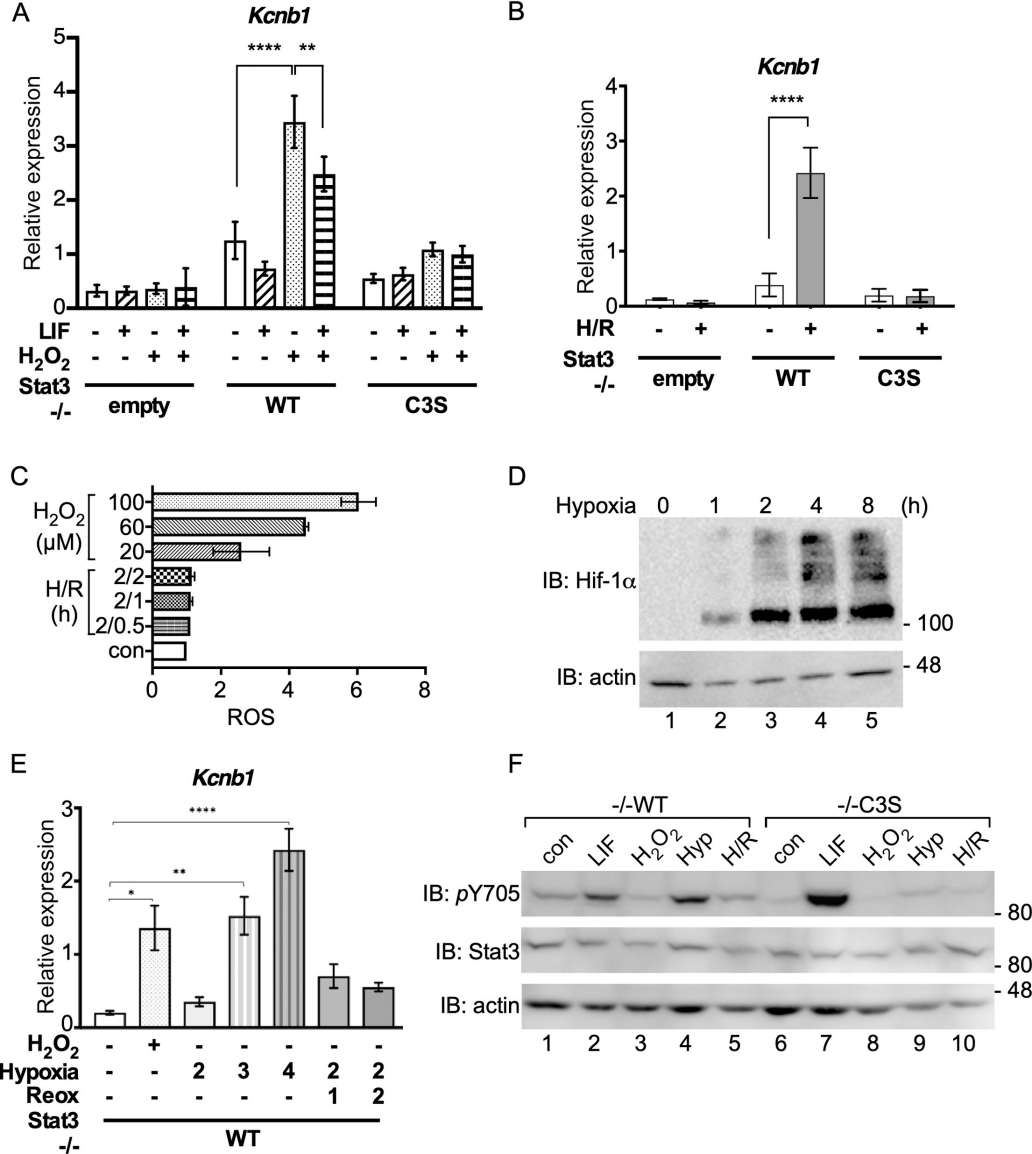
*Kcnb*1 expression in response to hypoxia/reoxygenation requires Stat3 oxidation. **A)** RNA isolated from MEFs untreated (-), treated with 10 ng ml^-1^ LIF for 30 mins and/or 100 μM H_2_O_2_ for 1 h (+) was enriched for mRNA and analysed for *Kcnb1* and *Hbs1l* expression by qRT-PCR. *Kcnb1* expression was normalised to *Hbs1l*. Data are expressed as mean ± SEM, n = 3. **B)** RNA isolated from MEFs untreated (-) or subjected to 2 h hypoxia (1% O_2_) and 2 h reoxygenation (H/R) was enriched for mRNA and analysed for *Kcnb1* and *Hbs1l* expression by qRT-PCR. *Kcnb1* expression was normalised to *Hbs1l*. Data are expressed as mean ± SEM, n = 3. **C)** -/- WT MEFs seeded to 96-well plates (1 x 10^4^ per well) and cultured under basal conditions overnight were stained with 100 μM DCFDA for 30 minutes and left to recover in full medium for 1 h when basal fluorescence was measured. Cells were treated with 0, 20 μM, 60 μM and 100 μM H_2_O_2_ or 2 h hypoxia followed by 30 mins, 1 h and 2 h reoxygenation, as indicated, before fluorescence measurement. Values are presented as means ± SEM from 3 independent determinations. **D)** -/-WT MEFs were exposed to hypoxia (1% O_2_) for the times indicated. Whole cell extracts were prepared and analysed for stabilisation of Hif-1α by SDS-PAGE and immunoblotting. **E)** -/- WT MEFs were subjected to normoxia (untreated, -), 100 μM H_2_O_2_, hypoxia for 2, 3 and 4 h and hypoxia for 2 h followed by 1 h and 2 h of reoxygenation (treated, +). qRT-PCR analysis was performed using mRNA enriched from total RNA. Expression of *Kcnb1* was normalised to expression of *Hbs1l*. Values are presented as mean ± SEM, n = 3 per group. *P<0,1, **P<0.01, ****P<0.0001. **F)** Whole cell lysates from -/-WT and -/-C3S MEFs untreated (con), treated with 10 ng ml^-1^ LIF for 30 mins (LIF), 100 μM H_2_O_2_ for 1 h (H_2_O_2_), 2 h hypoxia (1% O_2_; Hyp) or H/R as in (B) were analysed for Stat3 Y705 phosphorylation by SDS-PAGE and immunoblotting.

Although peroxide-responsive DE genes including *Kcnb1* were cytokine-unresponsive (Fig 3B–3E), co-treatment with LIF interfered significantly with peroxide-induced *Kcnb1* expression (Fig 5A). Because oxidative stress may contribute to tissue damage during ischaemia/reperfusion injury [2], we also tested the effect of hypoxia/ reoxygenation on *Kcnb1* expression. Two hours of hypoxia (1% O_2_) followed by 2 h of reoxygenation (H/R) stimulated *Kcnb1* expression in -/-WT cells but not in -/-empty or -/-C3S cells (Fig 5B). However, this response was unlikely due to oxidant stress because H/R did not induce a significant rise in intracellular ROS (Fig 5C). Stabilisation of Hif-1α appeared to plateau after 2 h of hypoxia in -/-WT cells (Fig 5D), but 2 h hypoxia caused no significant increase in *Kcnb1* expression (Fig 5E). After 3h hypoxia, *Kcnb1* expression was comparable to levels stimulated by peroxide (1 h) and increased with longer hypoxia treatment (Fig 5E). Thus, *Kcnb1* is responsive to oxidative stress and hypoxia [50] albeit over different time courses. The appearance of high molecular weight Hif-1α species at later times suggested that poly-ubiquitination of Hif-1α may continue or resume during chronic hypoxia [51].

Neither oxidative stress nor H/R increased Stat3 Y705 phosphorylation in -/-WT or -/-C3S cells, providing further evidence that Stat3-dependent *Kcnb1* expression in response to oxidative stress in -/-WT cells involves a non-canonical pathway. However, tyrosine phosphorylation Stat3 was detected in -/-WT cells after 2 h hypoxia but not in -/-C3S cells (Fig 5F), implying a mechanistic link between Stat3 oxidation and tyrosine phosphorylation during hypoxia.

**Fig 6:**
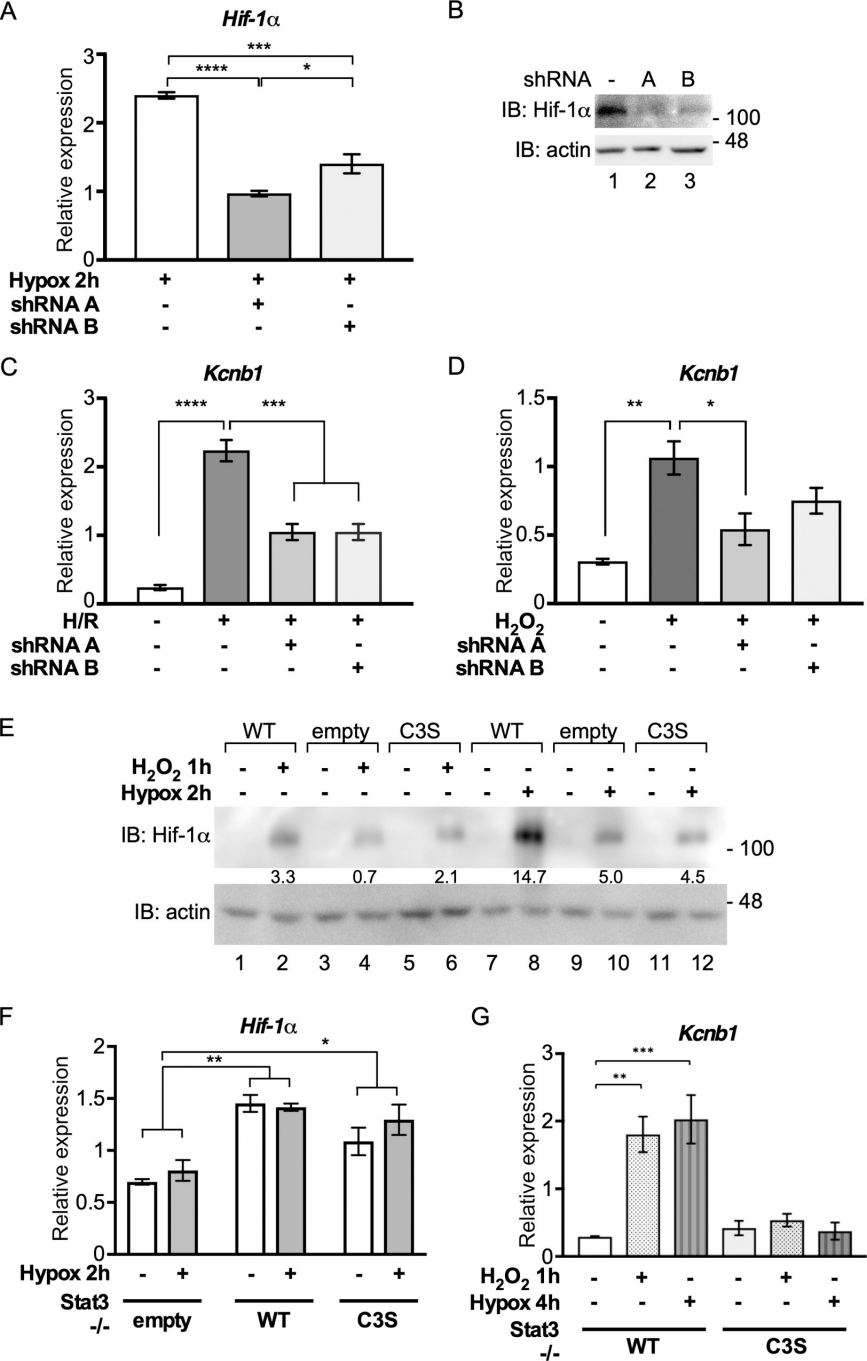
Stat3 and Hif-1α cooperate in the acute response to oxidative stress. **A)** *Hif-1*α expression in -/- WT MEFs transfected with shRNAs against *Hif-1*α and subjected to hypoxia for 2 h was monitored by qRT-PCR. *Hif-1*α was normalised against *Hbs1l*. Values are presented as mean ± SEM, n = 3 per group. *P<0,1, **P<0.01, ****P<0.0001. **B)** -/- WT MEFs transfected with shRNAs against *Hif-1*α were subjected to hypoxia for 2 h and Hif-1α protein levels were determined by SDS-PAGE and immunoblotting. **C)** -/-WT MEFs were transfected with shRNA vectors for *Hif-1*α or control vector and subjected to 2 h hypoxia (1% O_2_) and 2 h reoxygenation (H/R). Isolated RNA was enriched for mRNA and analysed for *Kcnb1* and *Hbs1l* expression by qRT-PCR. *Kcnb1* expression was normalised to *Hbs1l*. Data are expressed as mean ± SEM, n = 3. **D)** As in (C) except that cells were stimulated with 100 μM H_2_O_2_ for 1 h. **E)** MEFs were untreated (-), treated with 100 μM H_2_O_2_ for 1 h or hypoxia (1% O_2_) for 2 h, as indicated (+) and stabilisation of Hif-1α was determined by SDS-PAGE and immunoblotting. **F)** MEFs were untreated (-) or subjected to hypoxia for 2 h (+). Isolated RNA was enriched for mRNA and analysed for *Hif-1*α and *Hbs1l* expression by qRT-PCR. *Hif-1*α expression was normalised to *Hbs1l*. Data are expressed as mean ± SEM, n = 3. **G)** MEFs were untreated (-), treated with H_2_O_2_ for 1 h or subjected to hypoxia for 4 h, as indicated. Isolated RNA was enriched for mRNA and analysed for *Kcnb1* and *Hbs1l* expression by qRT-PCR. *Kcnb1* expression was normalised to *Hbs1l*. Data are expressed as mean ± SEM, n = 3.

We created two shRNA vectors for *Hif-1*α and confirmed that they depleted *Hif-1*α mRNA and protein in -/-WT cells (Fig 6A and 6B). Hif-1α depletion from -/-WT cells significantly decreased *Kcnb1* expression in response to H/R (Fig 6C) and to peroxide (Fig 6D), albeit to a lesser extent. This observation implicates Hif-1α in peroxide-stimulated *Kcnb1* expression. Furthermore, hypoxia (2 h) and peroxide treatment (1 h) both stabilised Hif-1α less efficiently in -/-C3S and -/-empty cells than in -/-WT MEFs (Fig 6E). Thus, oxidation of Stat3 contributes to stabilisation of Hif-1α.

Stat3 has been implicated in the regulation of *Hif-1*α transcription in response to hypoxia and mTORC1 signalling [46, 47]. We confirmed that *Hif-1*α mRNA levels were low in cells lacking Stat3 (-/-empty) but found no significant difference in *Hif-1*α mRNA levels between -/-WT and -/-C3S MEFs (Fig 6F). Nonetheless, *Kcnb1* was equally unresponsive to hypoxia and peroxide in -/-C3S cells (Fig 6G), indicating that the level of *Hif-1*α mRNA was not the limiting factor. In summary, these data reveal an inter-dependence between Stat3 oxidation and Hif-1α stabilisation in the regulation of *Kcnb1*, a gene that is acutely responsive to oxidative stress.

## Discussion

It is now recognised that oxidants generated by mitochondria or Nox enzymes modify discrete, intracellular targets that participate in signalling events to coordinate cell growth, differentiation and survival [5]. Here, we determined the impact of reversible oxidation of conserved cysteines within the DBD of Stat3 on global gene expression in MEFs under oxidative stress. We identified a cohort of 199 genes that were differentially expressed in response to Stat3 oxidation. Acutely up-regulated genes were linked to immune function and cell adhesion; down-regulated genes were associated with developmental processes and programmed cell death. Although consensus SIEs were present in the promoters of most DE genes, ChIP-seq provided no evidence of Stat3 DNA binding under basal conditions or in response to oxidative stress. Instead, our data point to an indirect activation mechanism in which Stat3 oxidation and Hif-1α stabilisation cooperate during acute up-regulation of genes in response to oxidative stress.

### Phenotypic change as a consequence of Stat3 cysteine substitutions

Cells that lacked a redox-sensitive Stat3 (i.e. -/-C3S and -/-empty MEFs) displayed flatter morphologies than -/-WT cells with pronounced peripheral actin fibres. This altered cell morphology correlated with significantly decreased rates of proliferation and migration, suggesting that redox signals normally transduced via Stat3 contribute to cell proliferation and migration while countermanding phenotypic change. Consistent with this notion is the elevated expression of genes linked to developmental processes seen in -/-C3S v -/-WT cells under basal conditions (no oxidative stress), along with the concomitant down-regulation of genes implicated in cell migration.

The cell lines also differed in their susceptibility to oxidative stress-induced cell death. The acute sensitivity of -/-empty cells to peroxide is consistent with a role for Stat3 in the protective response to oxidative stress [52]. In contrast, -/-C3S cells displayed a decreased sensitivity to peroxide (Fig 1H), for which two explanations seem plausible. Their increased survival could reflect hyperactivity of the canonical Stat3 pathway in -/-C3S cells, as revealed by elevated levels of Stat3 Y705 phosphorylation and *Socs3* expression (Fig 1D and 1E). Alternatively, survival could be due to impaired Stat3 redox signalling, as evidenced by the number of genes enriched in regulation of cell death and apoptosis that were dis-regulated in -/-C3S cells, suggesting that Stat3 oxidation is integral to intracellular ROS sensing. The potential to impact on either pathway in a mutually exclusive fashion suggests that Stat3 is a key arbiter of cell survival in response to oxidative stress.

### Discretized differential expression correlates with specific gene function

Multi-comparison analysis identified 3617 DE genes that segregated into 45 discrete clusters (S2A Fig), of which 10 were of interest as they encompassed those genes transcriptionally responsive to Stat3 oxidation (Fig 2D). GO term enrichment analysis partitioned these 199 genes strikingly into genes associated with membrane cell adhesion, immune function and ion transport that were up-regulated in -/-WT cells stimulated with peroxide (S4 Table), and genes associated with developmental processes that were down-regulated in peroxide-stimulated -/-WT cells (S5 Table).

Although participation of both redox signalling and Stat3 in inflammatory responses is well established [53, 54], the link between Stat3 oxidation and up-regulation of genes encoding immune regulators is new. For example, cluster 1,0,0,-1, which defines genes that are acutely sensitive to peroxide, is enriched for components of humoral and adaptive immune responses, such as *Foxj1* (6.6-fold), a regulator of T cell activation [55]; *Selplg* (10.4-fold), a promoter of myeloid cell recruitment [56]; *Hpx* (5.6-fold), a haem scavenger and suppressor of ROS-mediated macrophage activation [57]. Also up-regulated in peroxide-stimulated -/-WT cells are genes for Gpr15L (*2610528A11Rik*, 14.6-fold), recently implicated in T cell responses to epithelial damage [58] and *Nlrp3* (~30-fold), eponymous component of the ROS-inducible Nlrp3 inflammasome [59]. Of particular note is the acute up-regulation of *Il17f* (63-fold), in line with the central role of Stat3 in Th17-mediated pro-inflammatory responses and its implication in dermal disorders including HIES and psoriasis [60, 61]. Intriguingly, mutations in *Asprv1*, also acutely upregulated upon Stat3 oxidation (60-fold), are implicated in ichthyosis [62].

The connection between Stat3 oxidation and developmental processes is similarly compelling, as illustrated by the down-regulation in -/-WT cells of genes coding for growth factors and receptors (*Fgf7*, *Pgdfd*, *Pdgfrb*), matrix metalloproteases (*Adam19*, *Adamts12*, *Adamtsl3*), protein kinases (*Lrrk2*, *Syk*) and transcriptional regulators of other developmental genes (*Osr2*, *Esrrg*, *Bach2*, *Etv1*, *Pou3f3*, *Tfap2b*, *Trps1*, *Tshz2*, *Zfhx4*). Specifically, post-natal knockdown of *Esrrg* in mice resulted in cardiomyopathies and arrested mitochondrial maturation [63]; homozygous deletion of *Pou3f3* (Brn1) was associated with neonatal mortality and developmental defects in forebrain and kidney [64].

GO term enrichment analysis of individual clusters revealed deeper granularity of Stat3 oxidation-dependent expression pattern correlated with biological function. We focussed on the pruned version of total GO enrichment for individual clusters, obtained by removing semantically redundant GO terms. For example, down-regulated genes (in -/-WT cells) related to locomotion are selectively enriched in cluster -1,0,0,1, while down-regulated genes involved in PCD are predominantly in cluster -1,1,0,1 (Fig 3A and S6 Table). Thus, cysteine substitutions in Stat3 significantly increase expression of PCD genes under basal conditions, whereas for genes related to locomotion they only impair acute inhibition in response to peroxide. This implies that Stat3 can mediate distinct transcriptional responses across a range of peroxide concentrations.

### Does oxidative stress lead to changes in Stat3 chromatin occupation?

Oxidation of Stat3 *in vitro* compromised DNA binding and in cells induced the reversible formation of higher order complexes [18, 20]. Loss of DNA binding and participation of cysteines within the DBD in complex formation implied that Stat3 redox multimers do not interact with DNA, although this has yet to be formally established. It also remains unclear whether other proteins, e.g. Prx2, participate in redox multimers [20]. Accordingly, loss of gene expression in response to Stat3 oxidation was anticipated rather than the predominant, acute activation of genes that was observed. Validation by qRT–PCR of the substantial induction levels and lack of response to LIF confirmed the existence of a set of over 100 ROS-responsive Stat3 target genes with no clear mechanism for their activation.

A body of evidence indicates that unphosphorylated (Y705) Stat3 also interacts with DNA and could therefore transduce redox signals *in situ* [8, 65]. Alternatively, a non-canonical mode of DNA binding to purine-rich motifs has been described for mutant Stat3 alleles associated with HIES [31]. Our ChIP-seq data do not support either model. In unstimulated cells, the few Stat3 peaks that were apparent mapped to intergenic regions and a significant proportion of these sites bore AGG-like sequences. Although these sites could theoretically represent distal regulatory regions, they lack hallmarks of super-enhancers [66] and appear more likely to reflect background noise. Importantly, read peaks obtained from peroxide-treated cells (-/-WT and -/-C3S) were virtually identical to those in untreated controls and all contrasted starkly with data from LIF stimulated cells (S4F Fig).

ChIP-seq on LIF stimulated -/-WT MEFs identified 1088 read peaks associated with 927 genes with a striking correlation between read peaks and the presence of SIEs. Our gene set overlapped significantly with other Stat3 target gene sets, both amalgamated and data from murine ESCs [42], whereby differences due to experimental approach and cell type were expected [67]. In summary, we detected no Stat3 binding in unstimulated cells and pronounced cytokine stimulated Stat3 binding. There was virtually no difference between unstimulated and peroxide samples and the effect of peroxide on basal Stat3 binding was only apparent at the strongest canonical targets. Thus, peroxide-induced gene expression cannot be explained by Stat3 DNA binding. It remains possible that gene down-regulation in response to peroxide reflects loss of Stat3 binding at canonical targets, as evidenced earlier [18, 20], and ChIP-seq data provide evidence to support this model (Fig 4E). A ChIP-seq comparison between LIF alone and dual stimulation with LIF/ROS may reveal a stronger effect.

### A companion role for Hif-1a in Stat3-dependent, oxidant-responsive gene expression

Motif enrichment within gene regulatory regions highlighted candidate co-regulators of Stat3 target gene expression. Consensus TREs, AREs and HREs, binding sites for AP-1, Nrf2 and Hif-1α respectively, were enriched at DE gene promoters. These factors, as well as Stat3, have all been linked to the redox modulator APE/Ref-1 [68, 69]. Intriguingly, we also observed a significant co-association of consensus TREs and SIEs at LIF-inducible gene promoters. Several papers report functional interactions between Stat3 and Hif-1α [36, 70–74] and, like Stat3, Hif-1α is essential for ischaemic preconditioning in the heart [75].

The complex interrelationship between Stat3 and Hif-1α was manifest in the regulation of *Kcnb1*, a Stat3 target gene acutely responsive to peroxide stimulation (S2E Fig). Hypoxia also stimulated *Kcnb1* in -/-WT cells, albeit delayed with respect to peroxide stimulation (Fig 5E). Peroxide-induced *Kcnb1* expression required stabilisation of Hif-1α (Fig 6D) but in addition, Hif-1α stabilisation in response to peroxide was dependent on a redox-sensitive Stat3 (Fig 6E), as was hypoxia-inducible *Kcnb1* expression. Stat3 stimulates transcription of *Hif-1*α mRNA [46, 76] and significantly lower levels were present in -/-empty cells (Fig 6F). However, peroxide may also stabilise Hif-1α protein through inactivation of prolyl hydroxylases (PHDs) [77]. It is plausible that the Prx2-Stat3 relay participates in redox inactivation of PHDs and could act more rapidly than oxygen depletion to stabilise Hif-1α. Consensus HREs were found in 84/113 upregulated DE genes promoters. A Stat3-Hif-1α companion model may therefore not apply to all peroxide-responsive Stat3 target genes. Nonetheless, redox signalling provides Stat3 with ample scope for other liaisons.

Although our data imply an interdependency between oxidation of Stat3 and stabilisation of Hif-1α for the regulation of genes in response to oxidative stress, it remains to be established whether increased levels of Hif-1α protein could compensate for loss of Stat3 oxidation in terms gene expression, cell survival, proliferation or morphological change. Equally, how Stat3 target genes we identified here contribute individually to cellular responses to oxidative stress remains to be determined. In this regard, the availability of knockout mouse strains, e.g. for *Kcnb1* [78], may prove to be useful. Finally, our data suggest that aberrant gene regulation as a consequence of chronic Stat3 oxidation has the propensity to impact on diseases of the cardiovascular system, such as cardiac insufficiency or stroke, as well as those of the immune system, notably HIES [60]. The full clinical and therapeutic implications of these findings must await the outcome of further studies.

## Supporting information

S1 Fig **A)** MEFs were seeded in 96-well plates and cultured for times indicated. Cell proliferation was measured by MTT assay. **B)** Monolayers of the indicated MEFs growing under normal conditions were scratched with a 2 μl pipette tip and closure was monitored over 7 h by light microscopy. Images are representative of three independent experiments. **C)** MEFs were seeded and grown for 16 h under normal conditions. Cells were treated with 100 μM peroxide and levels of PCD were determined after 6 h using Muse Annexin V and dead cell assay kit, collecting 2000 cells per run.
(TIF)

S2 Fig **A)** Bar graphs showing an example for each of 45 unique patterns identified from 3617 differentially expressed genes. Y-axes throughout show normalised read counts. **B-H)** qRT-PCR analysis of genes indicated using total RNA from MEFs untreated (-) or stimulated (+) with 100 μM peroxide for 1 h. In all cases expression was normalized to expression of *Hbs1l*. Data are expressed as mean ± SEM, n = 5.
(ZIP)

S3 Fig **A)** Expression patterns of Stat3 oxidation-responsive genes enriched in cell migration GO category. Blue and red outlines indicate up- or down-regulation of genes respectively in -/-WT cells between oxidative (Oxd) and basal (Bas) comparisons. Values on Y-axis represent normalised read counts. **B)** Expression patterns of Stat3 oxidation-responsive genes enriched in positive regulation of cell migration GO category. Blue and red outlines indicate up- or down-regulation of genes respectively in -/-WT cells between oxidative (Oxd) and basal (Bas) comparisons. Values on Y-axis represent normalised read counts. **C)** Expression patterns of Stat3 oxidation-responsive genes enriched in regulation of cell death and apoptotic process. Blue and red outlines indicate up- or down-regulation of genes respectively in -/-WT cells between oxidative (Oxd) and basal (Bas) comparisons. Values on Y-axis represent normalised read counts.
(TIF)

S4 Fig Validation of Stat3 antibody used in ChIP experiments. **A)** -/-WT MEFs were untreated (-) or stimulated with 10 ng ml^-1^ LIF for 1 h. Cells were treated with formaldehyde for 10 min at 37°C, DNA-protein adducts were isolated and sonicated. After sonication, immunoprecipitates were collected with anti-Stat3 or IgG antibodies and analysed by SDS-PAGE and immunoblotting. **B)** ChIP analysis of Stat3 binding to *Socs3* and *Fos* promoters in untreated -/-WT MEFs or after treatment with 10 ng ml^-1^ LIF for 1 h, 100 μM H_2_O_2_ for 1 h or both (LIF/H_2_O_2_). **C)** Gene reporter assays in -/-WT and -/-C3S MEFs with murine *Kcnb1* P1 and P2 promoter regions coupled to firefly luciferase. Cells seeded in 24-well plates were untreated (-) or stimulated with 100 μM H_2_O_2_ for 4 h (+) prior to harvest. **D)** Chromatin distribution of Stat3 ChIP read peaks from unstimulated -/-WT MEFs or cells treated with LIF or H_2_O_2_ (Oxd). **E)** Venn diagram showing relationships between amalgamated canonical [1–9], ESC [10], ChIP and DE199 Stat3 gene sets (S7 Table). **F)** Genome viewer alignment of Stat3 ChIP read peaks mapped to intergenic loci in -/-WT and -/-C3S MEFs under basal conditions (Bas) or stimulated with H_2_O_2_ (Oxd) or LIF.
(TIF)

S1 Table Mapping of high quality reads generated using Illumina Hiseq 2500 sequencing platform to mm10 reference genome using Tophat2 v2.0.10. (XLSX)

S2 Table Differential expression analysis results showing 3617 differentially expressed genes (FDR corrected P < 0.05 and -1<logFC>1) between comparisons of -/-WT vs -/-C3S cells and basal vs oxidation treatments. Gene regulation discretised into 1 (up regulated), -1 (down-regulated and 0 (un-regulated).
(XLSX)

S3 Table Differential gene regulation clustering analysis results showing 199 differentially expressed genes clustered into 10 patterns. (XLSX)

S4 Table GO enrichment results for the 113 up-regulated genes. (XLSX)

S5 Table GO enrichment results for the 86 down-regulated genes. (XLSX)

S6 Table GO enrichment analysis results showing biological processes and their log(p-values) enriched by differentially expressed genes in each Stat3 oxidation responsive pattern (S3 Table). Patterns that do not show any GO enrichment are eliminated here.
(XLSX)

S7 Table Stat3 gene sets used to generate Venn diagram in supplementary S4E Fig. (XLSX)

S8 Table Genes with SIE (Stat3) binding sites and their positions in 3 kb upstream region from ATG. (XLSX)

S9 Table Genes with HRE (Hif-1) and TRE (AP-1) binding sites and their positions in 3 kb upstream region from ATG. (XLSX)

S10 Table Genes with ARE (Nrf2) binding sites and their positions in 3 kb upstream region from ATG. (XLSX)

S1 File (DOCX)

S1 Raw images Raw image files. (ZIP)
