# Supplementary figures and images for: Stat3 oxidation-dependent regulation of gene expression impacts on developmental processes and involves cooperation with Hif-1α
Source: PLoS One. 2020 Dec 17;15(12):e0244255. doi: 10.1371/journal.pone.0244255 (PMC7746180; doi:10.1371/journal.pone.0244255)

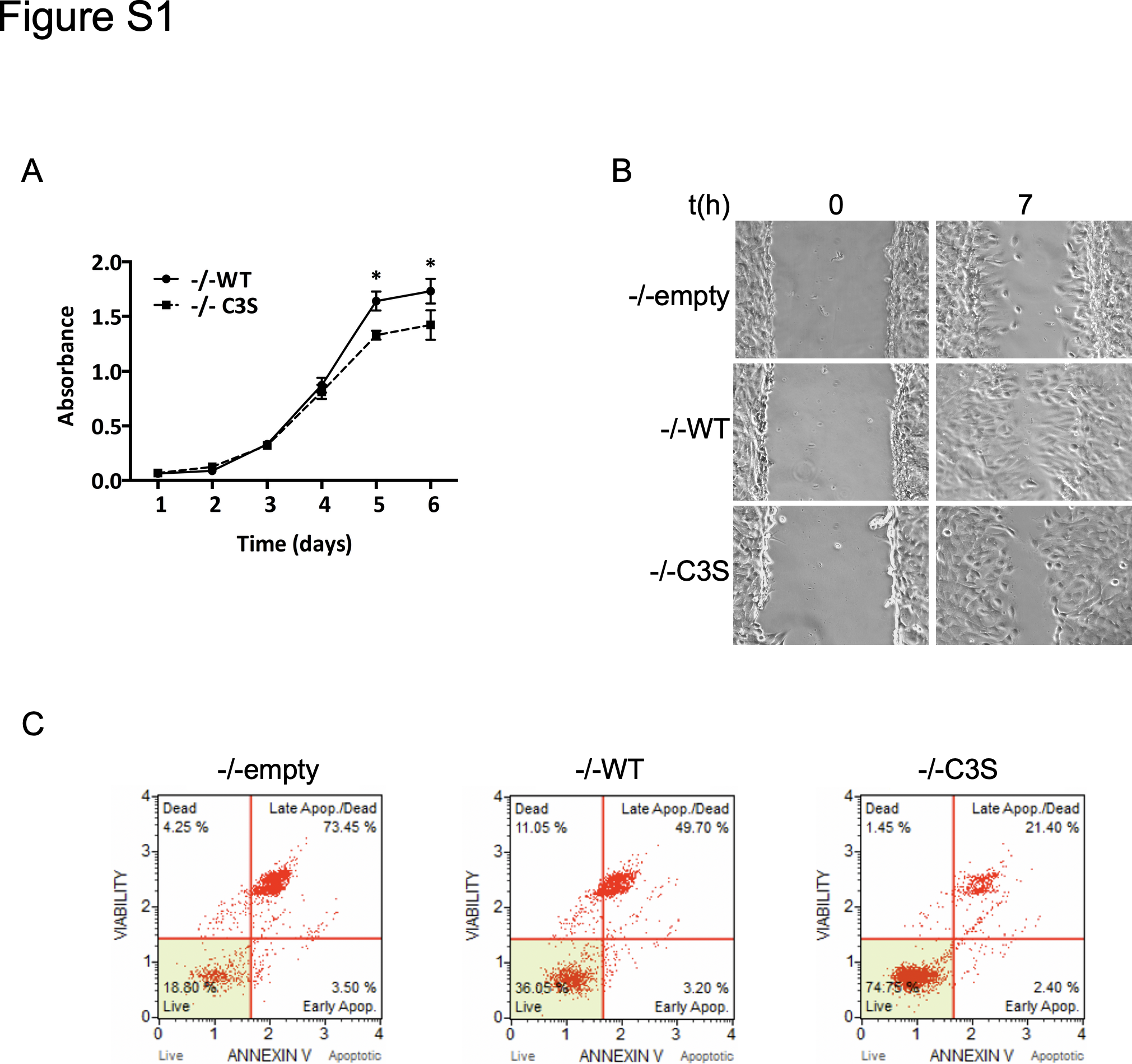

Supplement: S1 Fig — A) MEFs were seeded in 96-well plates and cultured for times indicated. Cell proliferation was measured by MTT assay. B) Monolayers of the indicated MEFs growing under normal conditions were scratched with a 2 μl pipette tip and closure was monitored over 7 h by light microscopy. Images are representative of three independent experiments. C) MEFs were seeded and grown for 16 h under normal conditions. Cells were treated with 100 μM peroxide and levels of PCD were determined after 6 h using Muse Annexin V and dead cell assay kit, collecting 2000 cells per run. (TIF) [file pone.0244255.s001.tif]

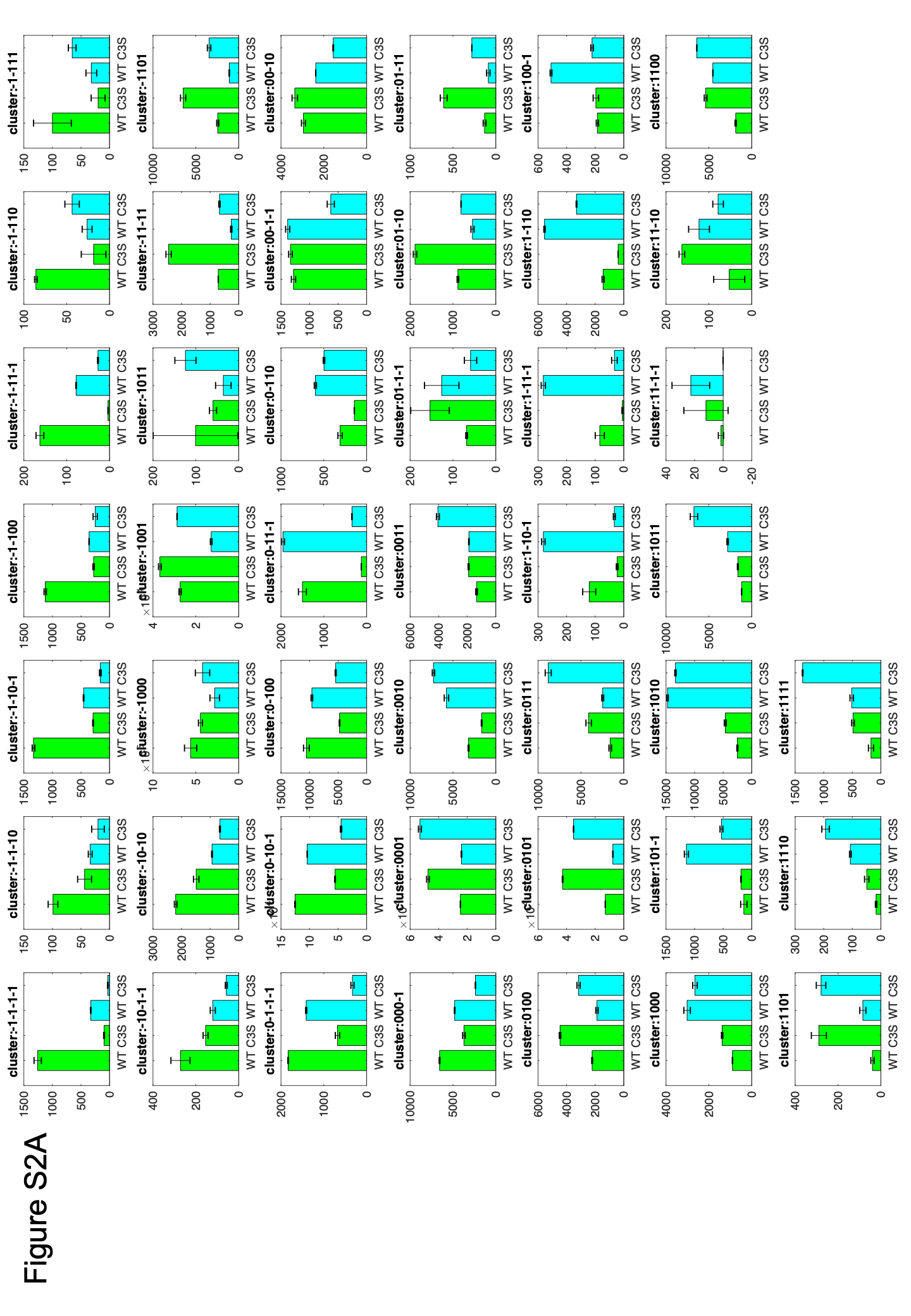

Supplement: S2 Fig — A) Bar graphs showing an example for each of 45 unique patterns identified from 3617 differentially expressed genes. Y-axes throughout show normalised read counts. B-H) qRT-PCR analysis of genes indicated using total RNA from MEFs untreated (-) or stimulated (+) with 100 μM peroxide for 1 h. In all cases expression was normalized to expression of Hbs1l. Data are expressed as mean ± SEM, n = 5. (ZIP) [file pone.0244255.s002.zip › S2A Fig.tif]

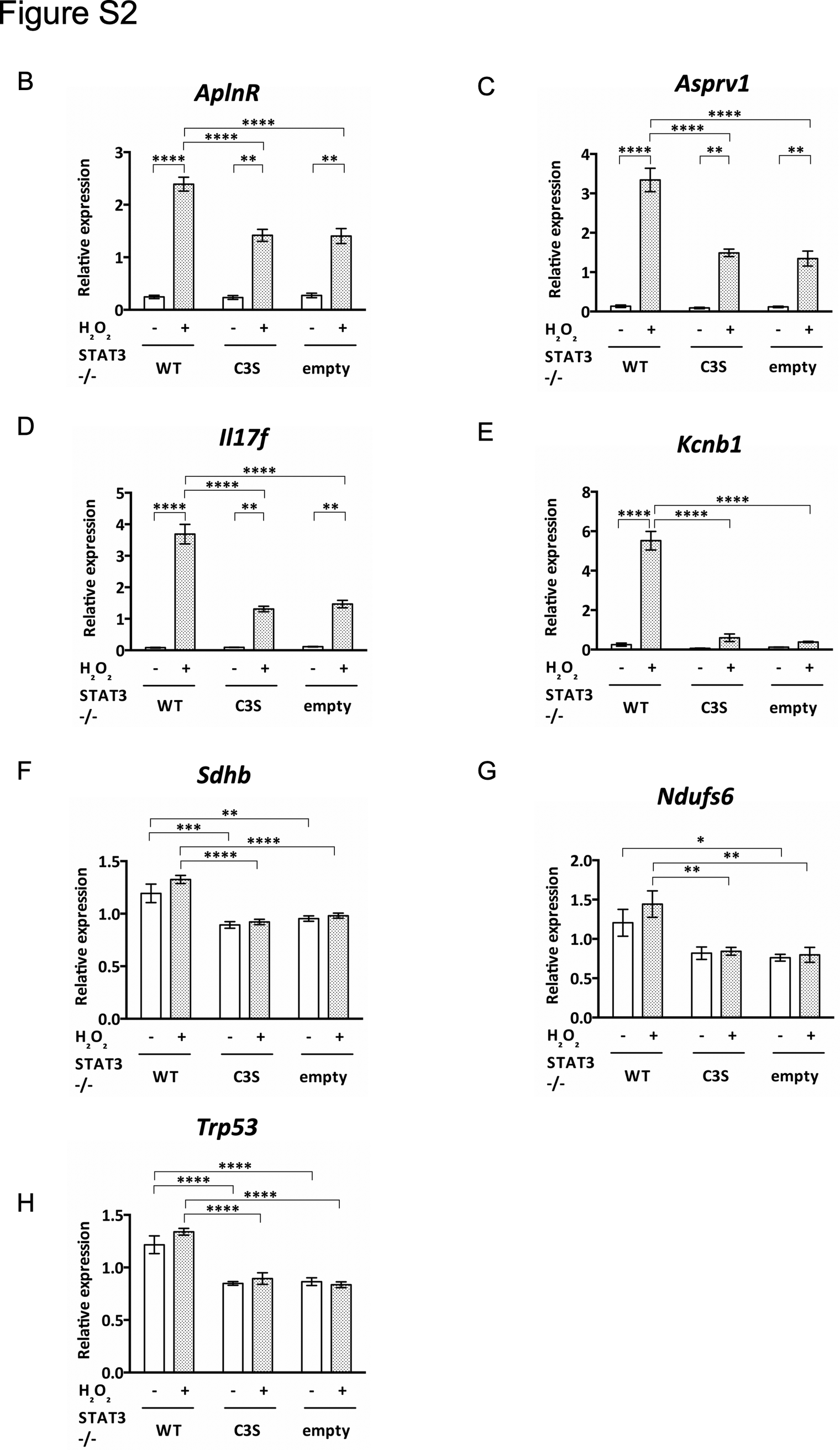

Supplement: S2 Fig — A) Bar graphs showing an example for each of 45 unique patterns identified from 3617 differentially expressed genes. Y-axes throughout show normalised read counts. B-H) qRT-PCR analysis of genes indicated using total RNA from MEFs untreated (-) or stimulated (+) with 100 μM peroxide for 1 h. In all cases expression was normalized to expression of Hbs1l. Data are expressed as mean ± SEM, n = 5. (ZIP) [file pone.0244255.s002.zip › S2B-H Fig.tif]

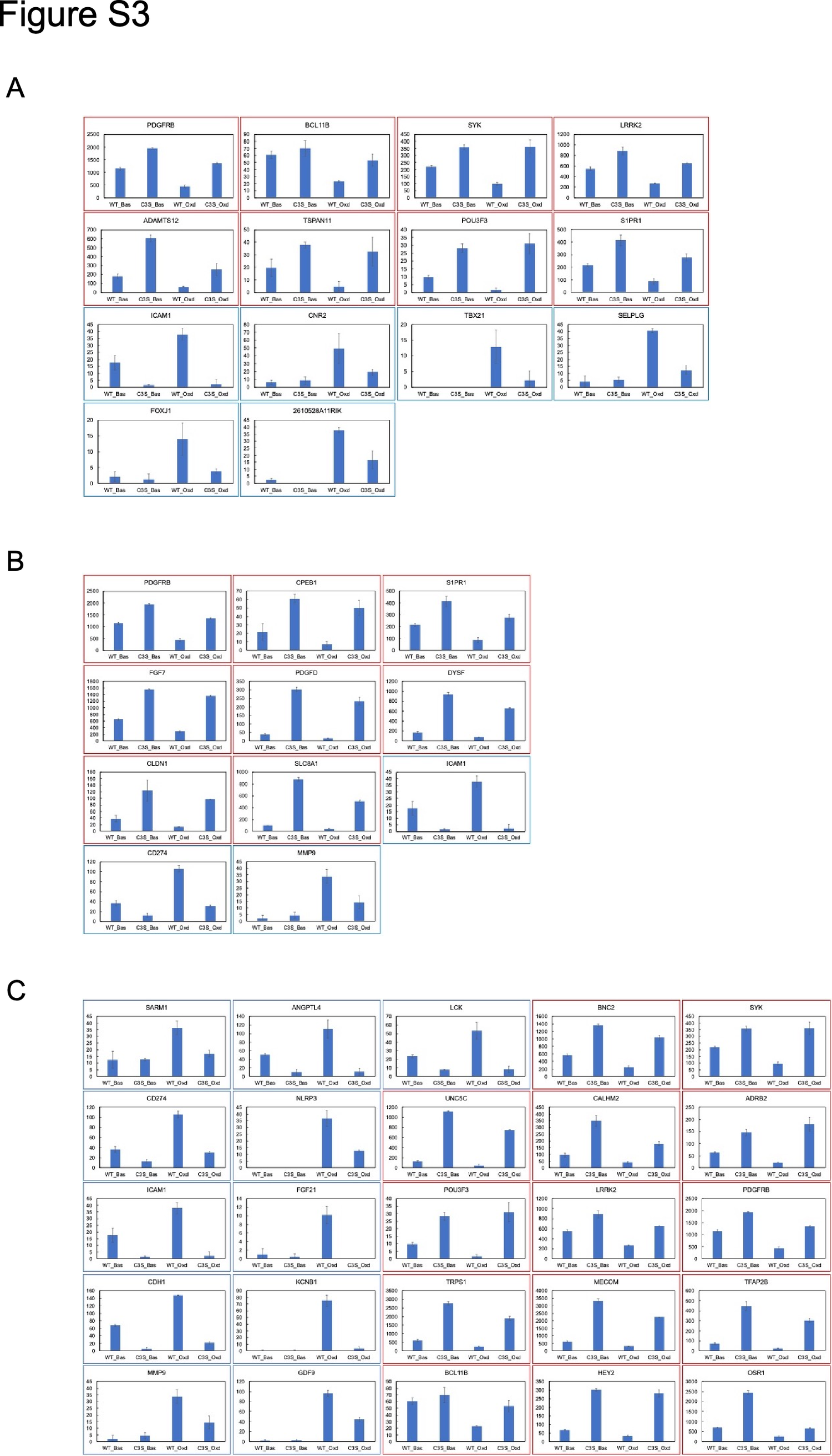

Supplement: S3 Fig — A) Expression patterns of Stat3 oxidation-responsive genes enriched in cell migration GO category. Blue and red outlines indicate up- or down-regulation of genes respectively in -/-WT cells between oxidative (Oxd) and basal (Bas) comparisons. Values on Y-axis represent normalised read counts. B) Expression patterns of Stat3 oxidation-responsive genes enriched in positive regulation of cell migration GO category. Blue and red outlines indicate up- or down-regulation of genes respectively in -/-WT cells between oxidative (Oxd) and basal (Bas) comparisons. Values on Y-axis represent normalised read counts. C) Expression patterns of Stat3 oxidation-responsive genes enriched in regulation of cell death and apoptotic process. Blue and red outlines indicate up- or down-regulation of genes respectively in -/-WT cells between oxidative (Oxd) and basal (Bas) comparisons. Values on Y-axis represent normalised read counts. (TIF) [file pone.0244255.s003.tif]

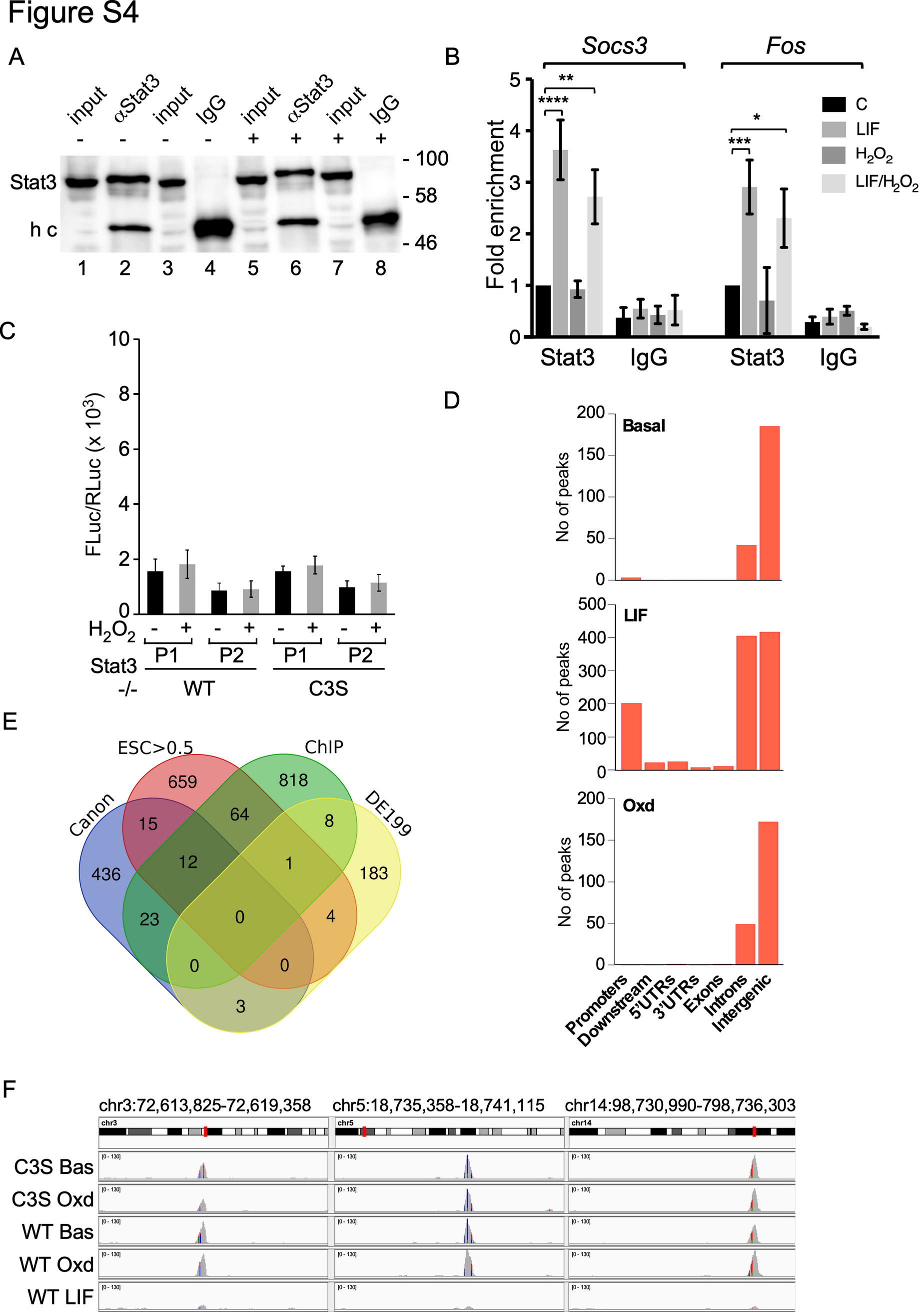

Supplement: S4 Fig — A) -/-WT MEFs were untreated (-) or stimulated with 10 ng ml-1 LIF for 1 h. Cells were treated with formaldehyde for 10 min at 37°C, DNA-protein adducts were isolated and sonicated. After sonication, immunoprecipitates were collected with anti-Stat3 or IgG antibodies and analysed by SDS-PAGE and immunoblotting. B) ChIP analysis of Stat3 binding to Socs3 and Fos promoters in untreated -/-WT MEFs or after treatment with 10 ng ml-1 LIF for 1 h, 100 μM H2O2 for 1 h or both (LIF/H2O2). C) Gene reporter assays in -/-WT and -/-C3S MEFs with murine Kcnb1 P1 and P2 promoter regions coupled to firefly luciferase. Cells seeded in 24-well plates were untreated (-) or stimulated with 100 μM H2O2 for 4 h (+) prior to harvest. D) Chromatin distribution of Stat3 ChIP read peaks from unstimulated -/-WT MEFs or cells treated with LIF or H2O2 (Oxd). E) Venn diagram showing relationships between amalgamated canonical [1–9], ESC [10], ChIP and DE199 Stat3 gene sets (S7 Table). F) Genome viewer alignment of Stat3 ChIP read peaks mapped to intergenic loci in -/-WT and -/-C3S MEFs under basal conditions (Bas) or stimulated with H2O2 (Oxd) or LIF. (TIF) [file pone.0244255.s004.tif]

Fig 1 panel B

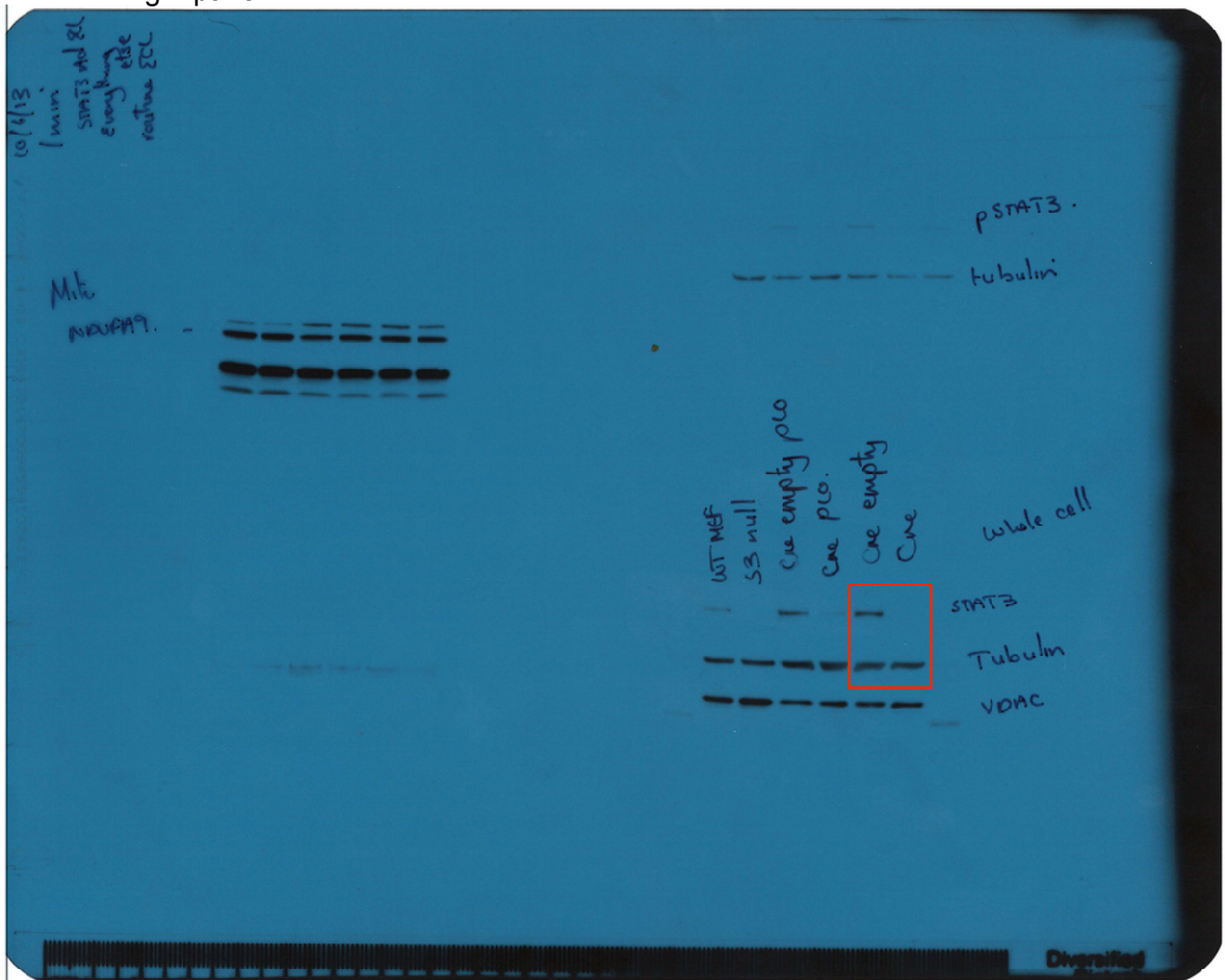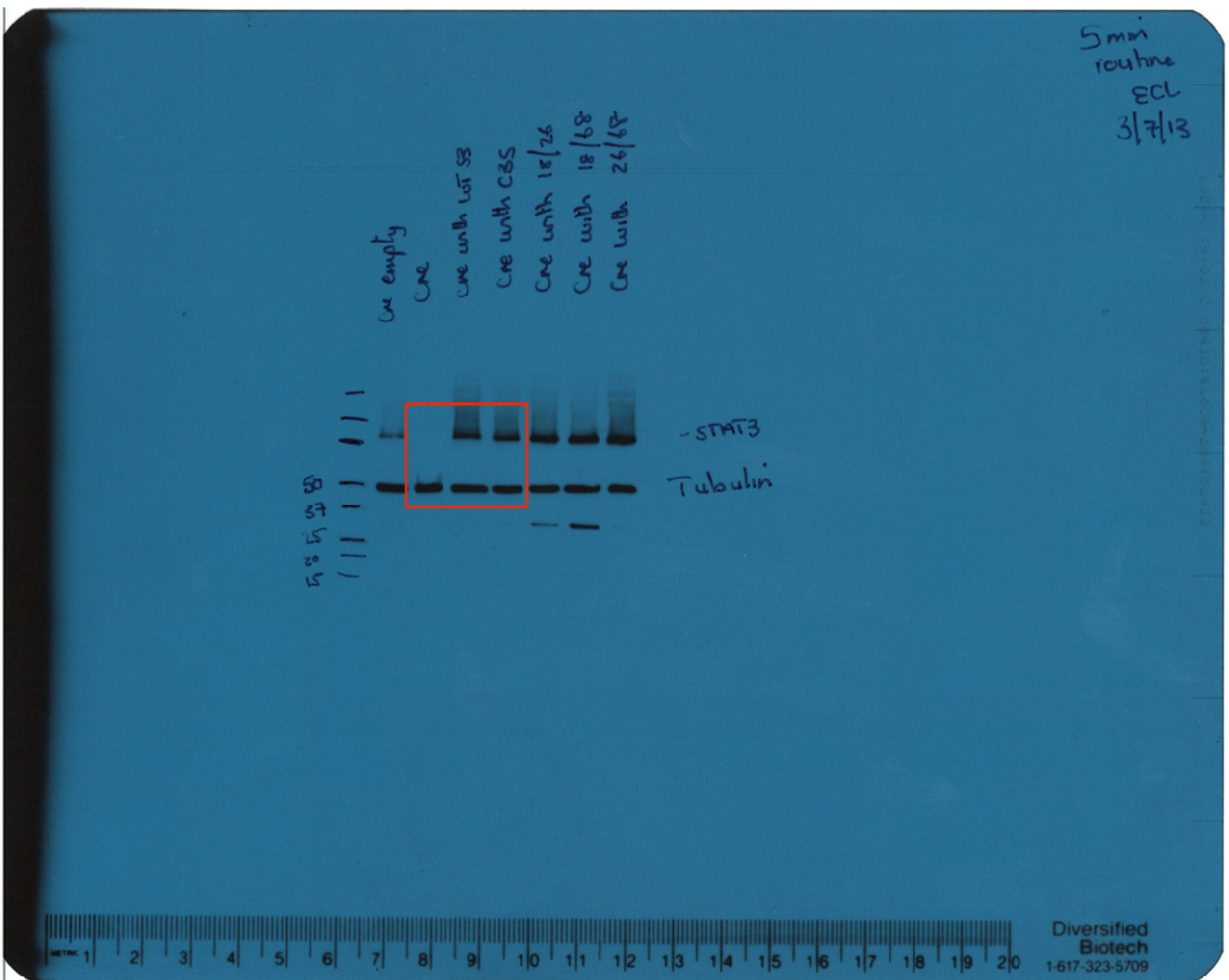

Fig 1 panel C

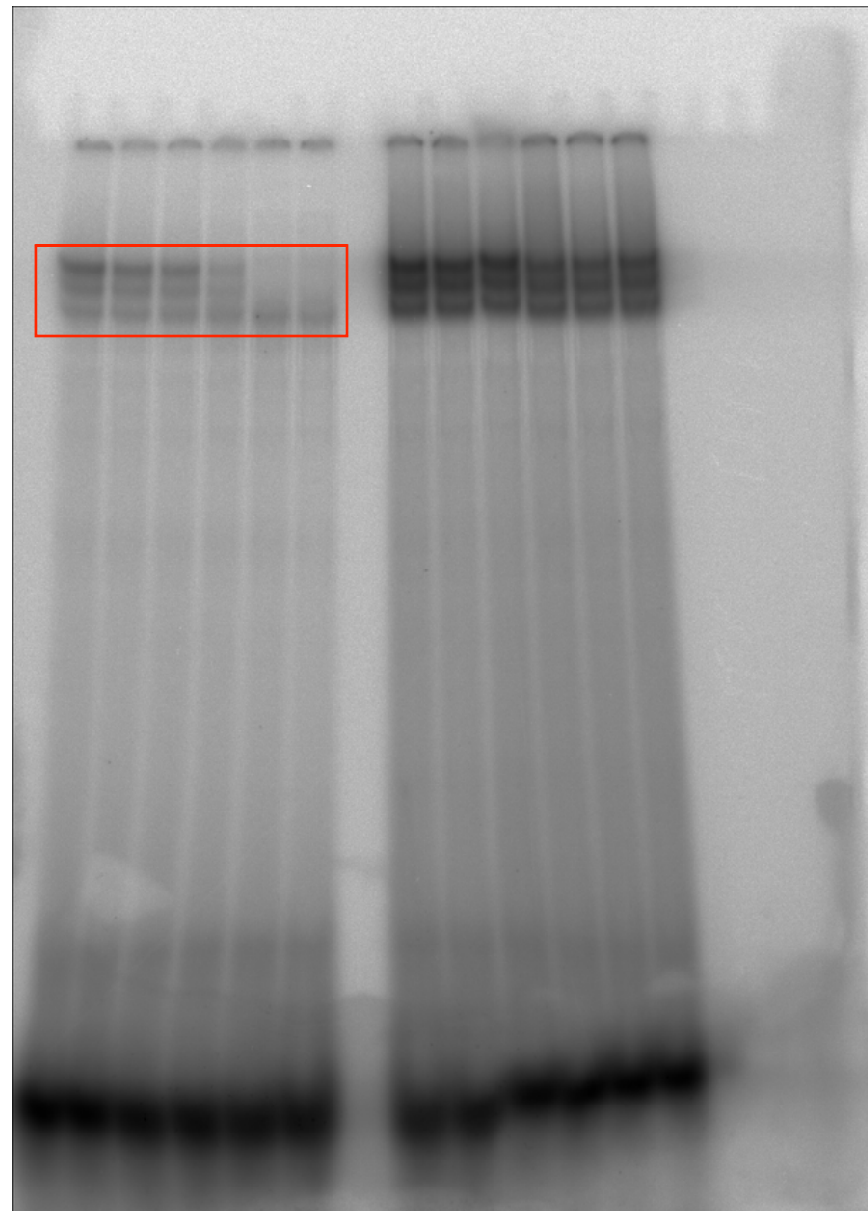

-/-WT

-/-C3S

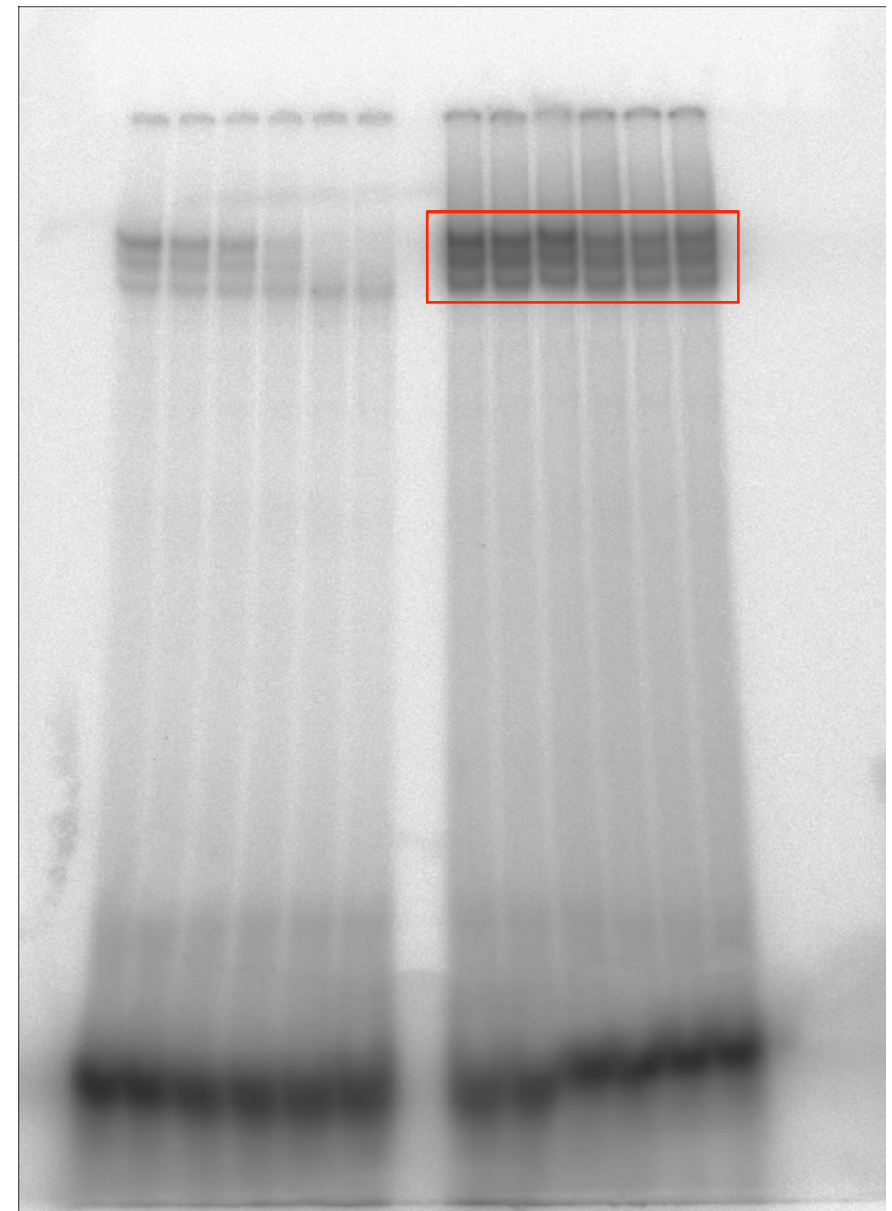

Figure 1 panel D

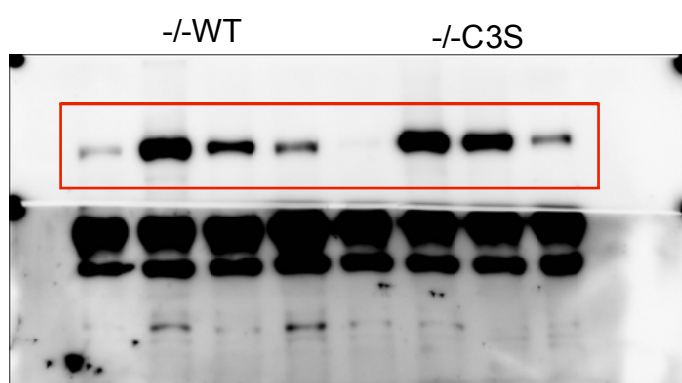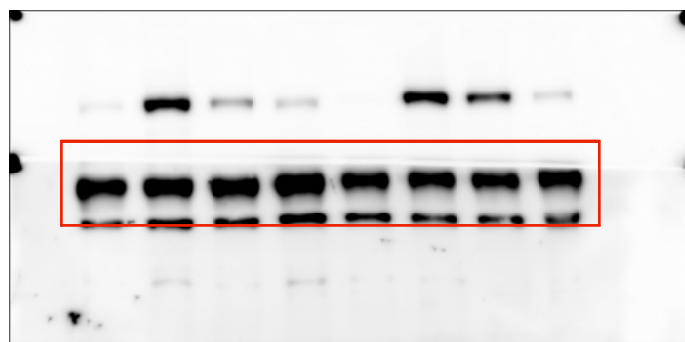

Fig 5 panel D

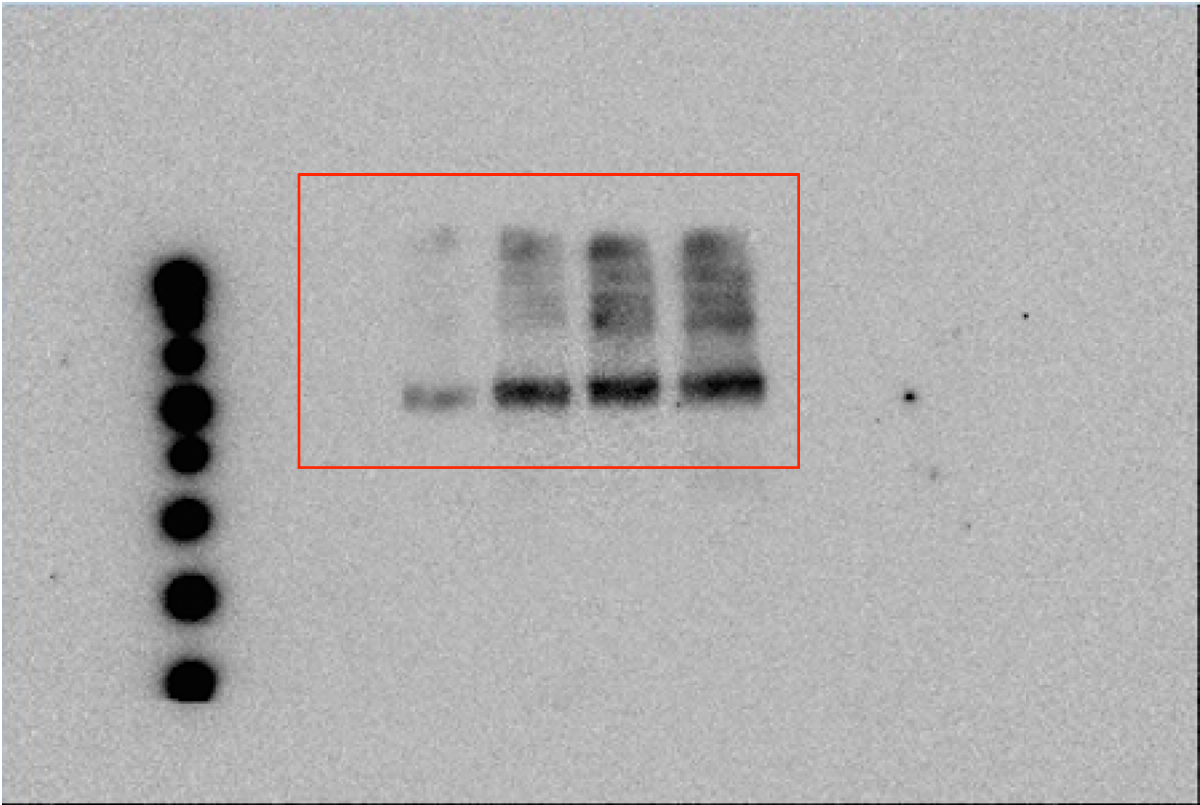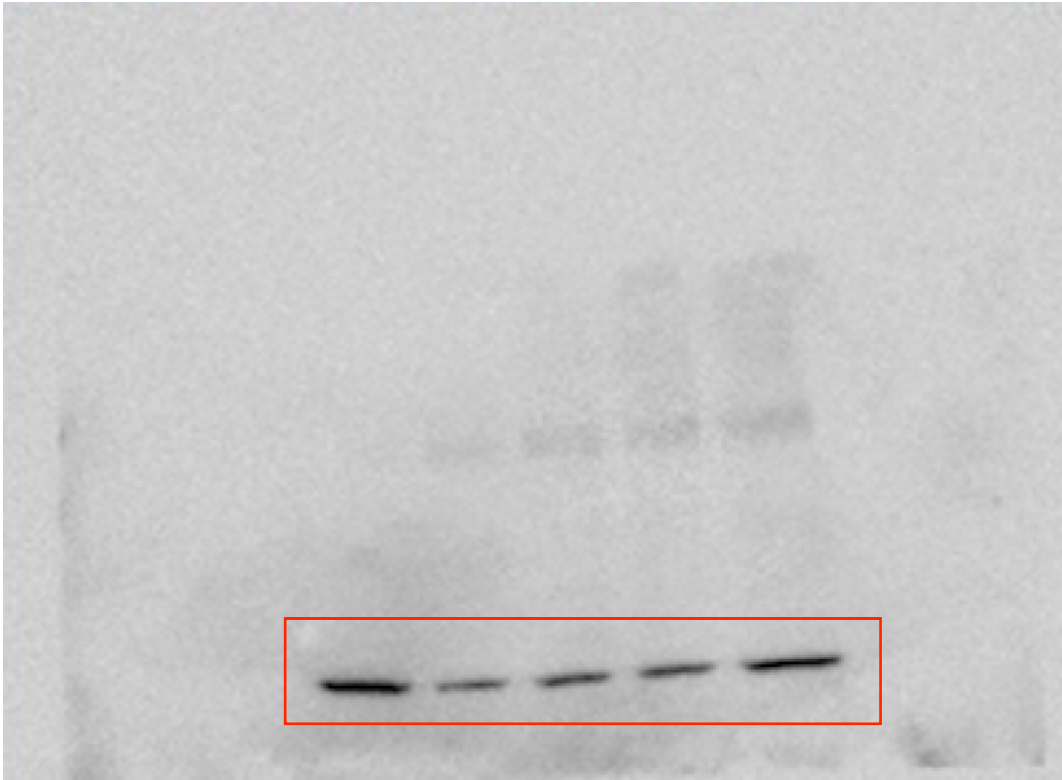

Fig 5 panel F

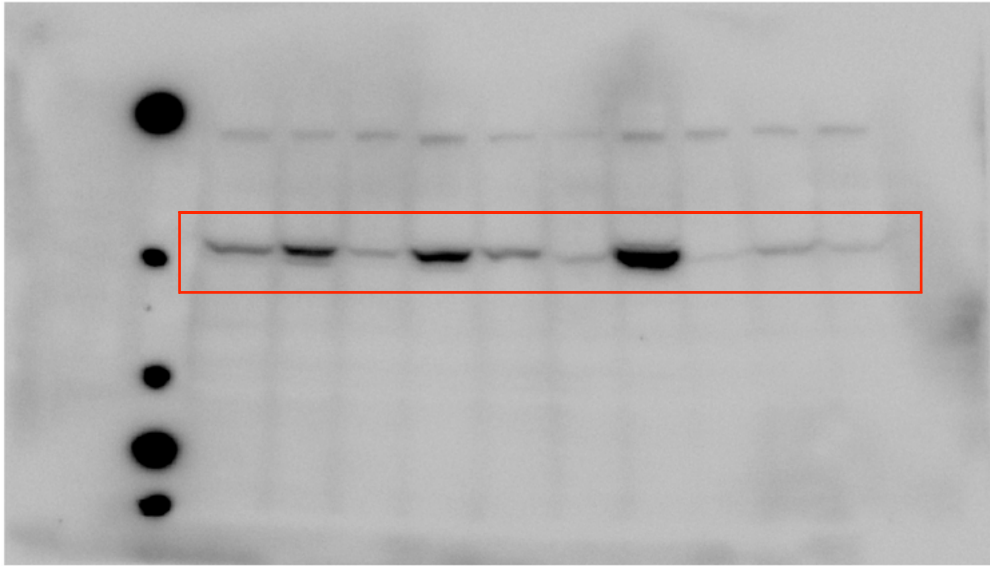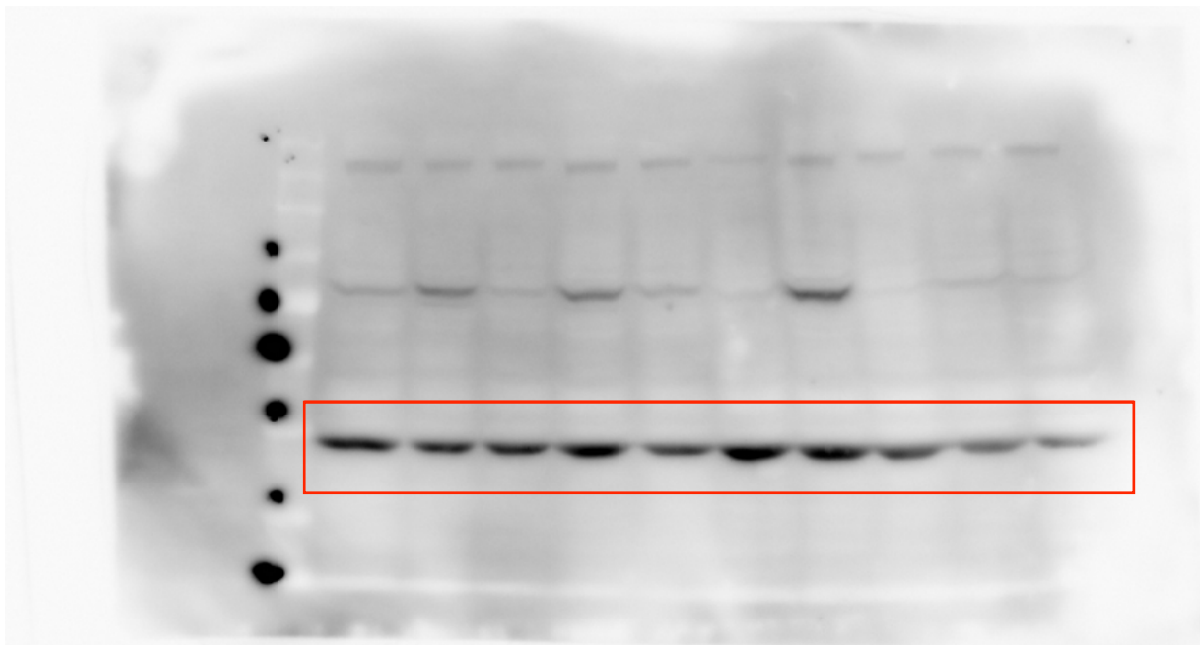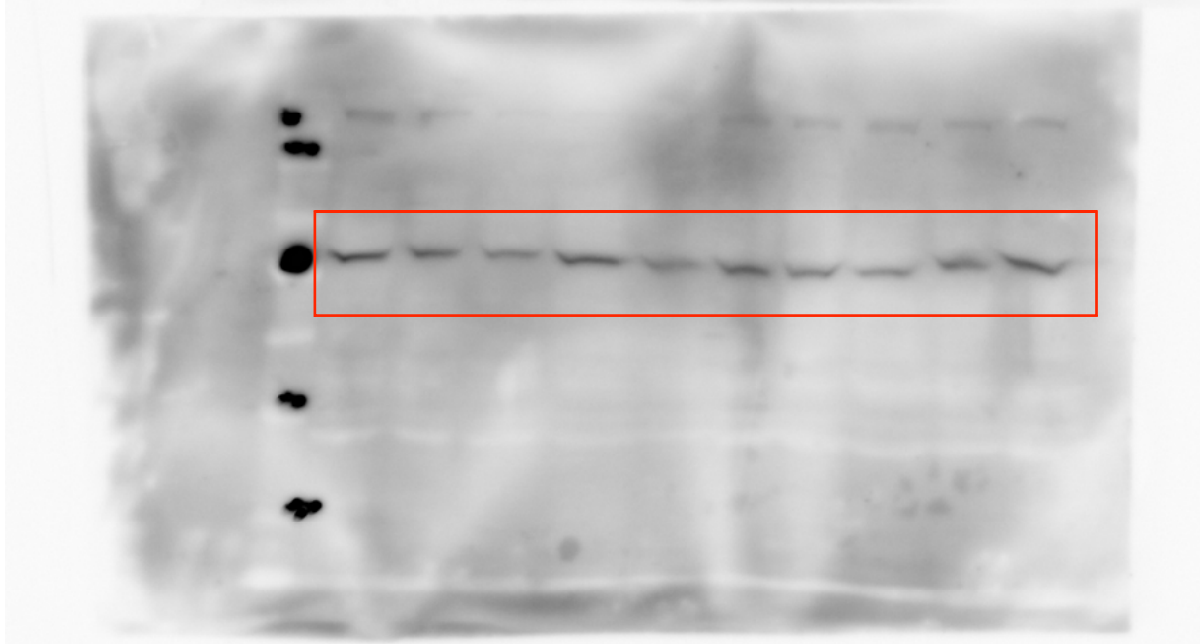

Fig 6 panel B

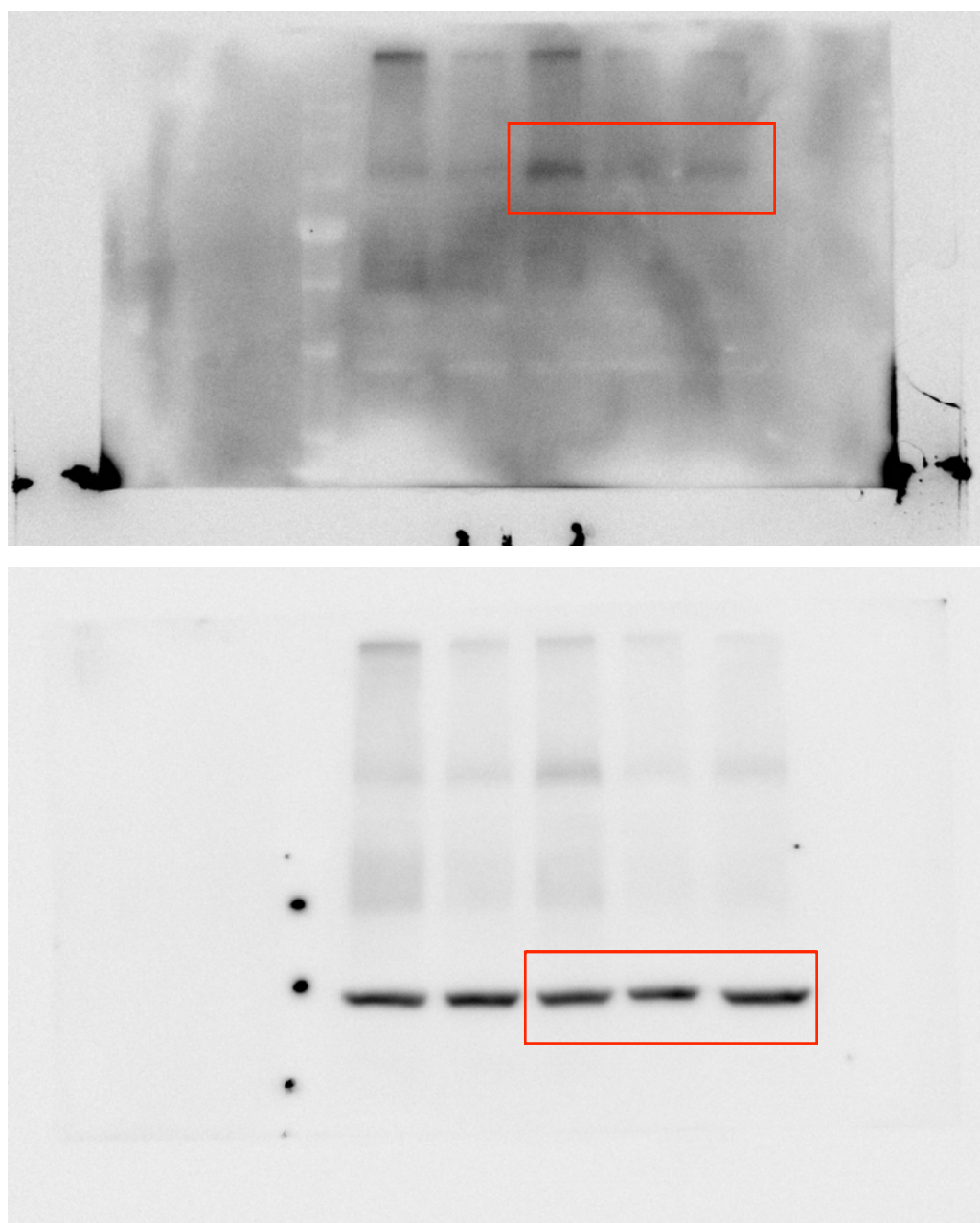

Fig 6 panel E

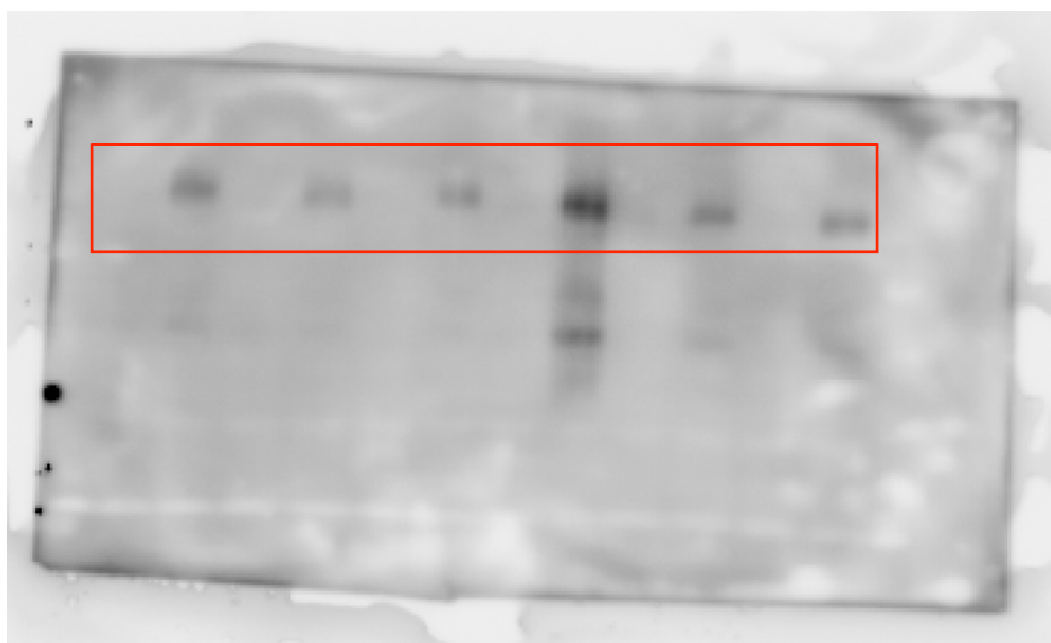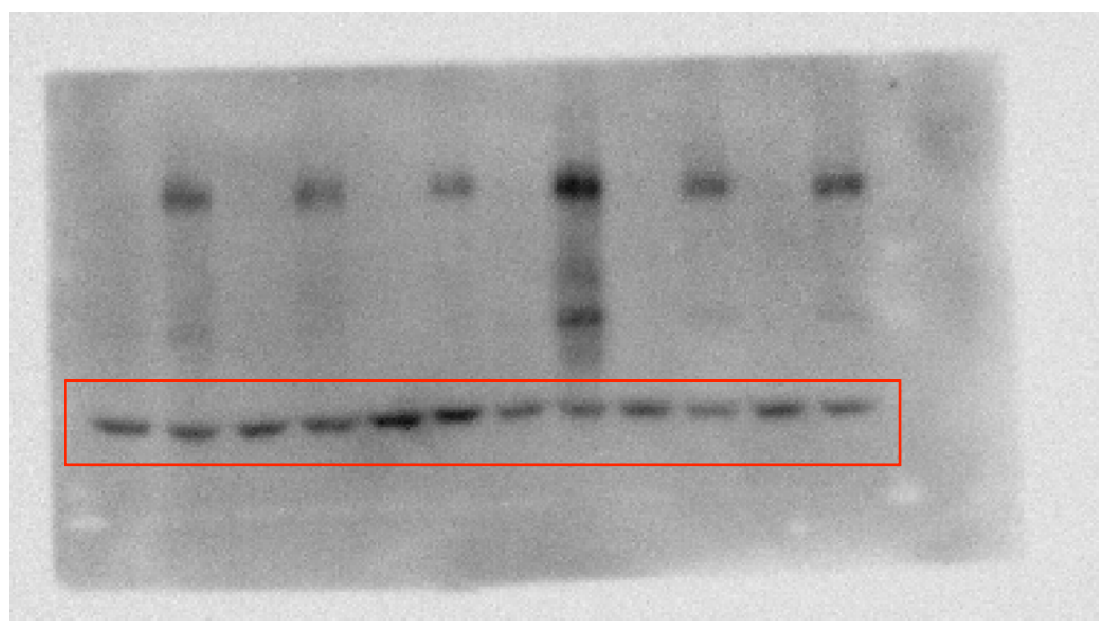

Supplement: S1 Raw images — (ZIP) [file pone.0244255.s016.zip › S1_raw_images.pdf]

S4 Fig panel A

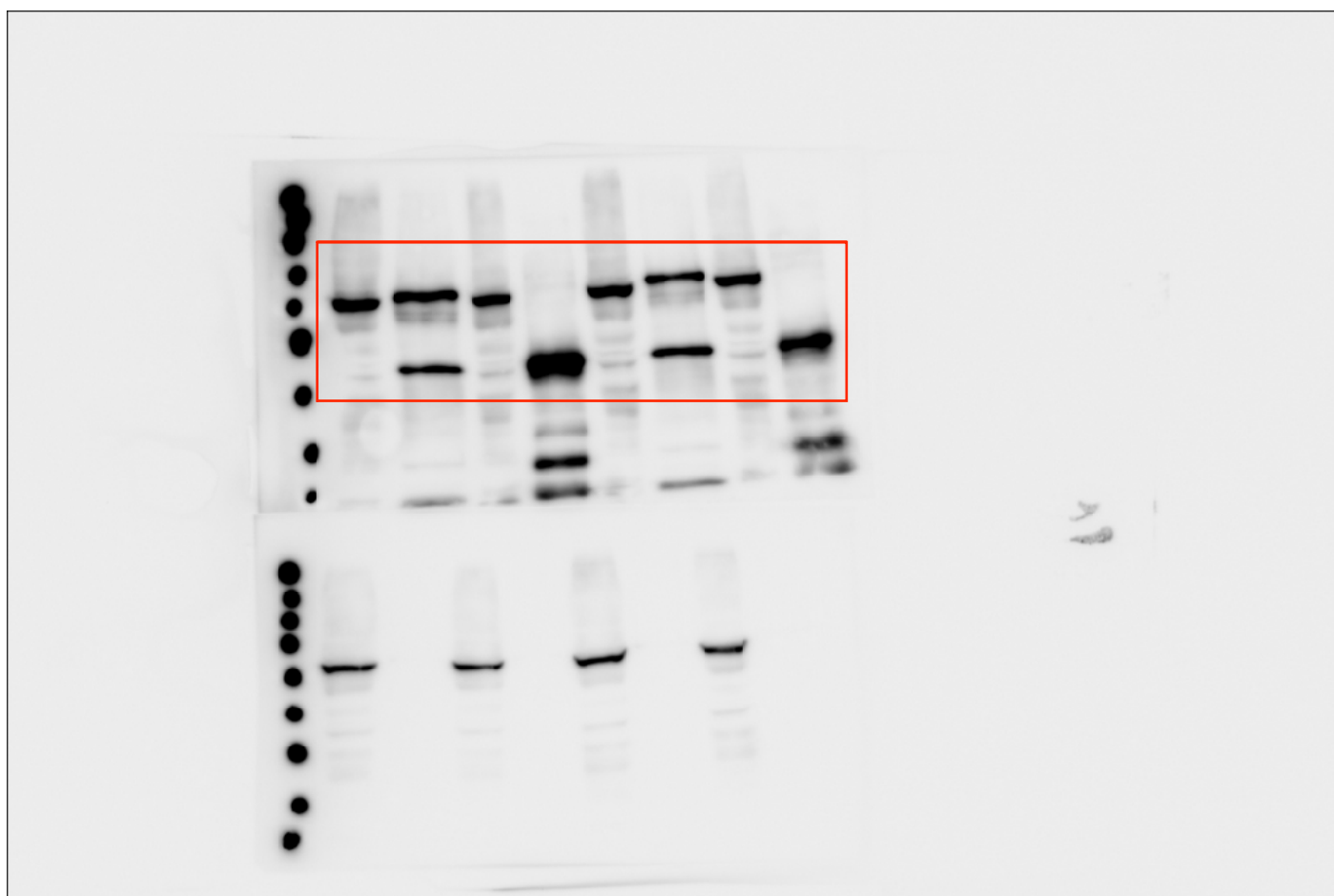

Supplement: S1 Raw images — (ZIP) [file pone.0244255.s016.zip › S4 Fig panel A.pdf]
