# Supplementary material for: Stat3 oxidation-dependent regulation of gene expression impacts on developmental processes and involves cooperation with Hif-1α
Source: PLoS One. 2020 Dec 17;15(12):e0244255. doi: 10.1371/journal.pone.0244255 (PMC7746180; doi:10.1371/journal.pone.0244255)
Supplement: S1 File — (DOCX) [file pone.0244255.s015.docx]

**S1 File**

**cDNA synthesis, library preparation and RNA sequencing**

RNA was enriched for mRNA, cDNA was synthesised and 8 barcoded directional RNAseq libraries were prepared using the NEBNext® Ultra™ Directional RNA Library Prep Kit (Illumina). Libraries were multiplexed into two lanes of Hiseq 2500 50bp rapid run to generate 220 million reads (Oxford Genomics Centre).

**Differential expression analysis**

Post sequencing, single fragment reads were trimmed against the sequencing library adaptor sequences using Scythe and low-quality bases were trimmed from the sequences using Sickle. Reads were aligned to mm10 reference genome using Tophat2 v2.0.10. Raw reads counts were normalised and differential expression analysis was performed using MATLAB’s RNAseq data analysis pipeline (<https://uk.mathworks.com/help/bioinfo/ug/identifying-differentially-expressed-genes-from-rna-seq-data.html>). Read count normalisation was performed in the following three steps: (i) generation of a pseudo-reference sample with counts obtained by considering the geometric mean of each gene across all samples (ii) estimation of size factors by taking the median of the ratios of observed counts to those of a pseudo-reference sample and (iii) transformation of observed counts to a common scale by dividing the observed counts in each sample by a corresponding size factor. Genes with a normalised read count of less than 10 in all samples combined were removed from further analyses. We inferred differential expression between two conditions for each comparison (-/-WT cells basal v oxidation treatments; -/- C3S cells basal v oxidation treatments; basal treatment -/-WT v -/-C3S and oxidation treatment -/-WT v -/-C3S) with a negative binomial model using ‘nbintest’ function with a null hypothesis that the two data samples come from distributions with equal means. The hypothesis testing was done by considering that the variance as the sum of shot noise term (mean) and a locally regressed non-parametric smooth function of the mean where counts are modelled according to the distribution proposed in [11]. The generated p-values were used corrected for multiple testing using Benjamini-Hochberg (BH) method [12]. Two-fold change (i.e. 1 logFC) and adjusted p-value < 0.05 cut-offs were used to obtain differentially expressed genes for further analysis.

**Differential gene regulation clustering and GO enrichment analysis**

We first discretised gene regulation information of each gene from aforementioned four pairwise comparisons into -1 (down-regulated), 1 (up-regulated) or 0 (un-regulated). For instance, in gene cluster -1,0,0,1, the first position attributes to comparison of oxidation v basal in -/-WT cells, the second position to comparison of -/-C3S v -/-WT cells under basal conditions, the third position to oxidation v basal in -/-C3S cells and the fourth position attributes to comparison of -/-C3S v -/-WT cells post oxidation (Fig 2C). In total 3617 genes were grouped into distinct 45 gene clusters, from which we then selected 10 clusters that showed Stat3 oxidation-responsive gene expression i.e. significant up- or down-regulation in control vs oxidation treatment in -/-WT cells with dis-regulation in -/- C3S cells and other comparisons. Next, we calculated functional enrichment for each of 10 clusters with the BiNGO tool [13] using hypergeometric tests and Benjamini-Hochberg multiple testing correction at FDR = 0.05 [12]. GO information and annotations for Mus Musculus were obtained from the GO database (www.geneontology.org; version downloaded on 08/11/2019). The whole annotation was used as the reference set for the GO enrichment analyses. Please note only 5 clusters resulted enrichment for at-least one GO term. The results are represented in Fig 3A and S6 Table.

**Promoter motif scan analysis**

We downloaded 3 kb FASTA sequences upstream of ‘ATG’ (on 23/02/2020) for 199 genes, which showed Stat3 oxidation-responsive expression, from mouse (GRCm38.p6) ensemble genome browser (https://www.ensembl.org/Mus_musculus/Info/Index). Highly conserved canonical motif sequences for Stat3 (‘TTCCNGGAA’), Hif-1 (‘RCGTG’), AP-1 (‘TGANTCA’) and Nrf2 (‘RTGANTCAGCA’) were obtained from previous reports [14-17]. We allowed up to two mismatches for Stat3 (either in the first or second half of sequence from ‘N’) and Nrf2 canonical sequences to generate combinations of motifs for scanning FASTA sequences.

**RNA extraction, cDNA synthesis and Taqman qRT-PCR**

MEFs untreated or treated with either 100 μM H_2_O_2_ for 1 h or 10 ng ml^-1^ LIF for 45 min were trypsinized and collected by centrifugation. Cells were homogenised and RNA purified using the Nucleospin RNA Kit (Macherey-Nagel). RNA was checked for quantity and quality using the Nanodrop 2000 (Thermo-Scientific) and RNA Nano BioAnalyzer chip (Agilent Technologies).

For gene sequences that do not allow primers to be designed across exon-exon junctions 1000 ng total RNA was enriched for mRNA using Dynabeads mRNA Purification Kit (Ambion by Life Technologies) prior to cDNA synthesis. For cDNA synthesis 500 ng of total RNA or mRNA eluted from Dynabeads was reverse transcribed using AffinityScript reverse transcriptase (Agilent Technologies) in a total reaction volume of 23 μl. Reactions were incubated at 25^O^C for 10 minutes, 50^0^C for 60 minutes and 70^0^C for 15 minutes to terminate the reaction. For genes with low expression (*Ndufs6*, *Kcnb1*, *Il17f, Asprv1,* *Aplnr*) a pre-amplification step was included prior to completion of Taqman assays.

Gene expression was quantified utilizing the relative standard curve method. Primers and probes were designed using Primer Express Software and synthesized by Eurofins Genomics (Germany). Gene expression data are presented as relative to *Hbs1l*.

**Primers and probes for Taqman qRT-PCR**

| **Gene** | **Forward primer** | **Reverse primer** | **TaqMan probe** |
| --- | --- | --- | --- |
| *Alkbh2* | 5-TCGTGAACAGGTACAAAGATGGTT-3 | 5-AGCCAGTTCTCGCTCGTCAT-3 | 5-CGACCACATCGGCGAGCACAGA-3 |
| *Ing4* | 5-GCGCACAAGTCCTGAGTATGG-3 | 5-ACAGGCATATCCAACACATCAGA-3 | 5-ATGCCCTCAGTGACCTTTGGCAGTGTC-3 |
| *Prdx2* | 5-ACCTGGCGTGGATCAATACC-3 | 5-CAAGCTTTTAGTCACGTCAGCAA-3 | 5-CTTGGGCCCCCTGAATATCCCTCTG-3 |
| *Sdhb* | 5-CGAAGATCTTGTAGAGAAGGCATCT-3 | 5-TGTGCACGCCAGAGTATTGC-3 | 5-CTCTTGCGCCATGAACATCAACGG-3 |
| *Trp53* | 5-CCACAGCGTGGTGGTACCT-3 | 5-TGTACTTGTAGTGGATGGTGGTATACTCA-3 | 5-AGCCACCCGAGGCCGGCT-3 |
| *Ndufs6* | 5-TTGTAGATCGTCAGAAAGAGGTGAAT-3 | 5- GCGGTGCTCCACCTCATT-3 | 5-TTGCCATTGATTTGATAGCACAACAGCC-3 |
| *Kcnb1* | 5-GCCATCATCTCCATCATGTTCA-3 | 5-GCTCTGTAGCTCAGGCAGTGTGT-3 | 5-TGTCCTCTCCACCATTGCCCTGTCAC-3 |
| *Alpnr* | 5-TTCTAGCTGTGCCTGTCATGGT-3 | 5-CACTGGATCTTGGTGCCATTT-3 | 5-TTCCGTTCCACAGACGCCTCGG-3 |
| *Il17f* | 5-TTCCAGAACCGCTCCAGTTC-3 | 5-CTGGGCCTCAGCGATCTCT-3 | 5-ATTACAACATCACTCGAGACCCCCACCG-3 |
| *Asprv1* | 5-AGCTGAAGGCCGAGTTTCTG-3 | 5-CGTCTGTGCCAATAATAGCCTCTT-3 | 5-TGGCCAACGCCAGCGCA-3 |
| *Hbs1l* | 5-CCCGAGAGCATGGCCTTT-3 | 5-TGCCAATTTACCTGATCCATCTT-3 | 5-TCCGATCTCTTGGAGTGACACAGCTTGC-3 |
| *Socs3* | 5-CCACCCTCCAGCATCTTTGT-3 | 5-CAGGCAGCTGGGTCACTTTC-3 | 5-ACTGTCAACGGCCACCTGGACTCCT-3 |

**Primers for chromatin immunoprecipitation**

| **Target** | **Forward primer** | **Reverse primer** |
| --- | --- | --- |
| *Socs3* | 5’-CGCGCACAGCCTTTCAGTG | 5’-TTTACCCGGCCAGTACGCC |
| *c-fos* | 5’-TCTGCCTTTCCCGCCTCCCC | 5’-GGCCGTGGAAACCTGCTGA |
| *Kcnb1 P1i* | 5’-TTGACCGAAGCTTTCTCCTTTA | 5’-CTTGCTCTGGCCTGATAGTT |
| *Kcnb1 P1ii* | 5’-AGAGGTCAGAGGGAGAGAAG | 5’-GTTCCTTTGCACTGCATCAC |
| *Kcnb1 P1iii* | 5’-ATGGGAAAGCCTTACCTTGG | 5’-TGTGCGGTGTTAAGGAGTTA |
| *Kcnb1 P2i* | 5’-GGAGGAGAAAGAGAGGAGGA | 5’-TCTGCACCTCCTTGTGC |
| *Kcnb1 P2ii* | 5’-CACTAAAGAGAGAAAGAAAGT | 5’-GTGCTGAAACTCCCTGCG |
| *Kcnb1 P2iii* | 5’-TGAGCCGCTTAAGCCTTG | 5’-CAGCGAACCGTACTGTGA |

**Chromatin immunoprecipitation and high-throughput sequencing (ChIP-seq)**

Stat3 -/-WT and -/-C3S MEFs were plated at 1.5 x 10^6^ cells per 10 cm dish in full medium. Cells were incubated in medium supplemented with 0.5% FBS over night, then treated with LIF 10 ng ml^-1^ for 45 min) or peroxide (100 µM for 1 h). After cross-linking with formaldehyde (1%, 10 min) cells were harvested in a pre-lysis buffer (125 mM glycine, 1 mM EDTA, 1 mM PMSF) collected by centrifugation and stored at -80^o^C.

Cells were lysed in 200 µl ice-cold lysis buffer (50 mM Tris-HCl pH 8.0, 1% SDS, 10 mM EDTA, 1 x protease inhibitor cocktail [Roche]) or 10 min, diluted to 300 µl and sonicated to produce 300-400 bp DNA fragments. Samples were cleared by centrifugation and 10% of each preparation was set aside as input fraction. For immunoprecipitations, samples were pre-cleared with agarose beads (2 h at 4^o^C), diluted and divided into 2 fractions to which were added either α-Stat3 antibody (124H6 Cell Signalling) or mouse IgG (MFCD00212351, Sigma) as negative control, and incubated on a wheel at 4^o^C over night. Immune complexes were collected by incubation with protein-A sepharose beads (GE Healthcare) for 1 h and centrifugation. Beads were washed serially in buffers of increasing ionic strength and detergent concentration, complexes were eluted from the beads in bicarbonate and incubated at 65^o^C for 6 h to reverse crosslinks. Samples were treated with proteinase K and DNA was purified using a GeneJet purification kit.

ChIP-Seq libraries were prepared using the NEBNext Ultra DNA library Prep Kit for Illumina (NEB; E7370S) and NEBNext Multiplex Oligos for Illumina (NEB; E7335, E7710, E7730), following instructions in the application note: Low input ChIP-Seq using the NEBNext Ultra Library Prep Kit for Illumina (<https://international.neb.com/-/media/catalog/application-notes/low-input-chipseq-using-the-nebnext-ultra-library-prep-kit-for-illumina-e7442.pdf?rev=91023778031446d88ebc26b53d3b8b83)>. The input volume of sheared ChIP DNA was 27.5 µl and the workflow included the recommended size-selection step and 14 cycles of library amplification. Libraries were quantified using the Qubit Fluorometer and the Qubit dsDNA HS Assay Kit (ThermoFisher Scientific; Q32854) and library fragment-size distribution was assessed using the Agilent TapeStation 4200 and the High Sensitivity D1000- ScreenTape Assay (Agilent; 5067-5584, 5067-5585). Libraries were pooled in equimolar amounts and the final library pool was quantified using the KAPA Library Quantification Kit for Illumina Platforms (Roche; KK4824). Libraries were sequenced on the Illumina NextSeq 500 using a NextSeq 500 High Output v2.5 150 cycle kit (Illumina; 20024907) to generate over 20 million pairs of 75-bp paired-end reads per sample.

**Amalgamation of canonical Stat3 target genes**

Genes identified as Stat3-regulated in [1-9] were collected, gene names were converted to official murine gene symbols and duplicates were removed yielding a canonical Stat3 gene set (771 genes, see S7 Table).

**Statistical analyses used to compare gene sets.**

Hypergeometric tests completed using Phyper or GeneProf were used to compare Stat3 oxidation genes to canonical Stat3 gene targets or Stat3 targets identified via ChIP-Seq. A comparison of canonical Stat3 gene targets and Stat3 targets identified via ChIP-Seq was also performed. The population size used for each of these tests was 23,499, which equates to the number of genes in the UCSC RefGene file for annotation of mm10.

**Literature cited in supplementary information**

1. Paz K, Socci ND, van Nimwegen E, Viale A, Darnell JE. Transformation fingerprint: induced STAT3-C, v-Src and Ha-Ras cause small initial changes but similar established profiles in mRNA. Oncogene. 2004;23(52):8455-63.

2. Azare J, Leslie K, Al-Ahmadie H, Gerald W, Weinreb PH, Violette SM, et al. Constitutively activated Stat3 induces tumorigenesis and enhances cell motility of prostate epithelial cells through integrin beta 6. Mol Cell Biol. 2007;27(12):4444-53.

3. Dechow TN, Pedranzini L, Leitch A, Leslie K, Gerald WL, Linkov I, et al. Requirement of matrix metalloproteinase-9 for the transformation of human mammary epithelial cells by Stat3-C. Proc Natl Acad Sci USA. 2004;101(29):10602-7.

4. Clarkson RW, Boland MP, Kritikou EA, Lee JM, Freeman TC, Tiffen PG, et al. The genes induced by signal transducer and activators of transcription (STAT)3 and STAT5 in mammary epithelial cells define the roles of these STATs in mammary development. Mol Endocrinol. 2006;20(3):675-85.

5. Snyder M, Huang XY, Zhang JJ. Identification of novel direct Stat3 target genes for control of growth and differentiation. J Biol Chem. 2008;283(7):3791-8.

6. Alvarez JV, Febbo PG, Ramaswamy S, Loda M, Richardson A, Frank DA. Identification of a genetic signature of activated signal transducer and activator of transcription 3 in human tumors. Cancer Res. 2005;65(12):5054-62.

7. Demaria M, Giorgi C, Lebiedzinska M, Esposito G, D'Angeli L, Bartoli A, et al. A STAT3-mediated metabolic switch is involved in tumour transformation and STAT3 addiction. Aging. 2010;2(11):823-42.

8. Sekkaï D, Gruel G, Herry M, Moucadel V, Constantinescu SN, Albagli O, et al. Microarray analysis of LIF/Stat3 transcriptional targets in embryonic stem cells. Stem Cells. 2005;23(10):1634-42.

9. Vallania F, Schiavone D, Dewilde S, Pupo E, Garbay S, Calogero R, et al. Genome-wide discovery of functional transcription factor binding sites by comparative genomics: the case of Stat3. Proc Natl Acad Sci USA. 2009;106(13):5117-22. doi: 10.1073/pnas.0900473106.

10. Chen X, Xu H, Yuan P, Fang F, Huss M, Vega VB, et al. Integration of external signaling pathways with the core transcriptional network in embryonic stem cells. Cell. 2008;133(6):1106-17. doi: 10.1016/j.cell.2008.04.043.

11. Anders S, Huber W. Differential expression analysis for sequence count data. Genome Biol. 2010;11:R106. doi: 10.1186/gb-2010-11-10-r106.

12. Benjamini Y, Hochberg Y. Controlling the False Discovery Rate: A Practical and Powerful Approach to Multiple Testing. J R Statist Soc B. 1995;57(1):289-300.

13. Maere S, Heymans K, Kuiper M. BiNGO: A Cytoscape Plugin to Assess Overrepresentation of Gene Ontology Categories in Biological Networks. Bioinformatics. 2005;21(16):3448-9. doi: 10.1093/bioinformatics/bti551.

14. Ehret GB, Reichenbach P, Schindler U, Horvath CM, Fritz S, Nabholz M, et al. DNA binding specificity of different STAT proteins: Comparison of in vitro specificity with natural target sites. J Biol Chem. 2001;276(9):6675-88.

15. Schödel J, Oikonomopoulos S, Ragoussis J, Pugh CW, Ratcliffe PJ, Mole DR. High-resolution genome-wide mapping of HIF-binding sites by ChIP-seq. Blood. 2011;117(23):e207-17. doi: 10.1182/blood-2010-10-314427.

16. Angel P, Hattori K, Smeal T, Karin M. The jun proto-oncogene is positively autoregulated by its product, Jun/AP-1. Cell. 1988;55(5):875-85. doi: 10.1016/0092-8674(88)90143-2.

17. Malhotra D, Portales-Casamar E, Singh A, Srivastava S, Arenillas D, Happel C, et al. Global mapping of binding sites for Nrf2 identifies novel targets in cell survival response through ChIP-Seq profiling and network analysis. Nucleic Acids Res. 2010;38(17):5718-34. doi: 10.1093/nar/gkq212.
